# Intravaginal poly-(D, L-lactic-*co*-glycolic acid)-(polyethylene glycol) drug-delivery nanoparticles induce pro-inflammatory responses with *Candida albicans* infection in a mouse model

**DOI:** 10.1371/journal.pone.0240789

**Published:** 2020-10-22

**Authors:** Taslima T. Lina, Shemedia J. Johnson, R. Doug Wagner

**Affiliations:** 1 Microbiology Division, National Center for Toxicological Research, U.S. FDA, Jefferson, Arkansas, United States of America; 2 Office of Regulatory and Risk Management, National Center for Toxicological Research, U.S. FDA, Jefferson, Arkansas, United States of America; Xiangtan University, CHINA

## Abstract

In a recent study, using an in vitro model to study intravaginal nanoparticle exposure during yeast infections, we demonstrated that *C*. *albicans* exposure suppressed apoptotic gene expression and induced oxidative stress and pyroptosis in vaginal epithelial cells. The mucous-penetrating drug delivery nanoparticles made from poly-(D, L-lactic-*co*-glycolic acid)-(polyethylene glycol) induced cytotoxicity by activating apoptosis, endoplasmic reticulum (ER) stress, oxidative stress, and DNA damage repair responses alone and, in some cases with *C*. *albicans*. In the current study we evaluated the effects of fluorescently-labelled nanoparticles in CBA/J mice challenged intravaginally for two hours followed by intravaginal challenge with *C*. *albicans* for 18 hours. Nanoparticle treatment increased systemic translocation of *C*. *albicans* threefold in the heart. *C*. *albicans* also increased systemic distribution of the nanoparticles fivefold in the heart. Flow cytometric assays showed co-localization of the nanoparticles with epithelial cells, macrophages and dendritic cells. Nanoparticle-treated, *C*. *albicans*-infected mice exhibited induction of autophagy, ER stress, apoptosis, and inflammatory serum cytokines. *C*. *albicans* infection was associated with pyroptosis and suppressed expression of ER stress and apoptosis-related genes. Induction of apoptosis during nanoparticle treatment and in nanoparticle-treated-*C*. *albicans* infected mice was observed as DNA damage responses, mitochondrial depolarization and (Poly [ADP-Ribose] Polymerase) cleavage. *C*. *albicans* infection was associated with increased mRNA expression of anti-apoptotic genes. Both *C*. *albicans* infection and nanoparticle treatment showed enhanced chemoattraction of dendritic cells and polymorphonuclear cells to factors in vaginal washings in a chemotaxis assay. This study shows that both intravaginal treatment of mice with the nanoparticles and infection with *C*. *albicans* induce cytotoxic and inflammatory responses. *C*. *albicans* also suppressed cell apoptosis. These results clarify our understanding of how nanoparticles modulate host cellular responses during *C*. *albicans* infection and will be applicable for future research and development of intravaginal nanomedicines.

## Introduction

Vulvovaginal candidiasis is an opportunistic fungal infection predominantly caused by overgrowth of *Candida albicans* that colonizes the vagina. The infection induces a strong inflammatory response in localized vaginal and vulvar epithelial tissues [1]. It remains the most prevalent organism in both mucosal and systemic yeast infections. The disease occasionally affects 75% of healthy women [2] and is a constant problem for a growing number of immunodeficient women. We recently showed that vaginal epithelial cells (VEC) in vitro contribute to inflammatory recruitment by secreting inflammatory cytokines after recognizing *C*. *albicans* through Nod-like receptors and a pyroptosis mechanism [1]. Though the innate immune response is required for clearance of *C*. *albicans*, the increased inflammation induced during the infection is also responsible for symptoms of vaginal candidiasis and morbidity of the disease [3]. Because of the inflammation associated with candidiasis, there is concern about the use of targeted nanoparticle-based drug delivery technology for intravaginal use, since these products could either improve treatment effectiveness or might aggravate the condition.

The development of controlled-release systems for drugs to mucosal surfaces such as those of the female reproductive tract is of widespread interest, especially for delivery of anti-infective drugs. The limited permeability of conventional drug delivery through the vaginal mucus barrier leads to their rapid clearance from the delivery site, often precluding effective drug therapies at non-toxic dosages [4]. Nanoparticles, with an average size below 100 nm, represent an alternative for prolonged drug delivery through the mucus barrier [1]. While numerous polymers have already been used in the production of nanoparticles, mucous-penetrating nanoparticles consisting of poly-(D, L-lactic-*co*-glycolic acid)-(polyethylene glycol) (PLGA-PEG) could improve targeting of microbicidal drugs for sexually transmitted diseases by intravaginal inoculation [5, 6]. Because of their unique physical and chemical properties, nanoparticles can interact with biological components and systems at the nanoscale and induce a variety of biological responses. Inorganic nanoparticles cause cytotoxicity by activating endoplasmic reticulum (ER) stress, mitochondrial depolarization, apoptosis, and autophagy [7–9]. Recently, we used an in vitro system (VK2 vaginal epithelial cells) to demonstrate that PLGA-PEG induced cytotoxicity and programmed cell death responses alone and during *C*. *albicans* infection [1]. After evaluating cell stress response pathways, we concluded that PLGA-PEG induced cytotoxicity by initiating endoplasmic reticulum stress and autophagy pathways, which led to mitochondrial depolarization and subsequent activation of apoptosis. Responses to *C*. *albicans* occurred through oxidative stress which induced pyroptosis. Pro-inflammatory cytokine production by the VEC resulted from challenge with either *C*. *albicans* or PLGA-PEG alone and in combination. The tendency of *C*. *albicans* to induce pyroptosis, but not apoptosis in that study was interesting. Different intracellular cytotoxicity responses of the cells to *C*. *difficile* and PLGA-PEG suggested that proinflammatory responses to these two factors could lead to exacerbation of vaginal candidiasis in women using the polymeric drug-delivery vehicle.

The mechanisms of PLGA-PEG nanoparticle toxicity have not been fully explored, especially in the context of an intact immune system. Currently, hazard characterization of nanoparticles is predominantly based on in vitro test systems. In this study we investigated the contribution of PLGA-PEG to cellular stress responses, e.g., autophagy, DNA damage, and apoptosis in an in vivo vaginal candidiasis murine model. This model has many similarities to humans regarding inflammatory infiltration of vulvovaginal tissues. Inflammatory cytokine responses and chemotaxis of bone marrow-derived dendritic cells (DC) and polymorphonuclear cells (PMN) were also analyzed using the same model. We evaluated the underlying signal transduction pathways modulated by the drug-delivery nanoparticles and *C*. *albicans* yeast infections as they relate to cellular stress and systemic inflammatory responses. We measured induction of cellular stress responses in autophagy and apoptosis, and production of inflammatory cytokines such as IL-1β, IL-6, and TNFα during PLGA-PEG treatment and *C*. *albicans* infection. Our study design illustrates the effect of PLGA-PEG on VEC and their role in induction of an inflammatory network and may provide insights to guide the development of protective measures for the application of these nanoparticles.

## Materials and methods

### Murine vaginal candidiasis model

A murine model of *C*. *albicans* vaginal infection was used to study the immunological effects of intravaginal inoculation of fluorescently labelled PLGA-PEG because mice have a vaginal tissue inflammatory cell recruitment response similar to humans [10, 11]. Female CBA/J mice were obtained from Jackson Laboratories at 8 weeks of age (Jackson Laboratories, Bar Harbor, ME, USA) and were used in this study with the approval of the National Center for Toxicological Research Institutional Animal Care and Use Committee. Sterile water and NIH-31 mouse chow were supplied ad libitum to the mice. Mice were pre-conditioned by subcutaneous injections of 0.05 mg 17β-estradiol valerate in sesame oil 72 hours prior to intravaginal instillation of 15 μL of 100 mg/mL PLGA-PEG nanoparticles suspended in phosphate buffered saline (PBS) for 2 hours followed by intravaginal challenge with 50 μL of 1 X 10^6^ CFU/mL virulent *C*. *albicans* strain B-311. Groups of six mice were treated with (1) PBS vehicle control, (2) *C*. *albicans*-challenged, (3) PLGA-PEG treated, and (4) PLGA-PEG-treated *C*. *albicans*-challenged. Eighteen hours after challenge, mice were euthanized and vaginal washings, blood, vaginal tissues, and internal organ samples were collected for analyses.

### Microbial strains and human cell lines

*Candida albicans* B311 (ATCC 32354) was grown in Sabouraud's dextrose broth (Thermo Fisher, Houston, TX, USA) at 37°C, as previously described [12]. Jurkat, Clone E6-1 (ATCC TIB-152) cells (American Type Culture Collection, Manassas, VA, USA) were grown in RPMI 1640 medium supplemented with 10% fetal bovine serum, 100 IU/mL Penicillin, 100 μg/mL Streptomycin, 0.25 μg/mL Amphotericin B, 10 mM HEPES, and 2 mM L-glutamine (RPMI) at 37°C in an atmosphere with 10% CO_2._ Cell lines and media were tested for *Mycoplasma* spp. contamination using a polymerase chain reaction (PCR) assay (Mycoplasma Detection Kit, Southern Biotech, Inc., Birmingham, AL, USA). Procedures involving the use of human cell lines were reviewed and approved by the U.S. Food and Drug Administration (FDA) Institutional Review Board. Procedures involving biosafety level 2 microorganisms were reviewed and approved by the FDA Institutional Biosafety Committee.

### Nanomaterials

PEG_3400_-conjugated PLGA-PEG nanoparticles (approximately 50–100 nm diameter) covalently labelled with the fluorophore FKB-350 (excitation wavelength 352 nm, emission wavelength 408 nm), for high content imaging and fluorescence activated cell sorter (FACS) analysis were synthesized to our specifications by Phosphorex, Inc. (Hopkinton, MA, USA), as described previously [1]. These nanoparticles passed tests for sterility and low endotoxin content. Solutions and media used in this study were assayed for endotoxin concentrations with a *Limulus* amebocyte lysate assay (Thermo Fisher).

### Detection of nanoparticle accumulation in mouse cells

The cervix and urethra were removed from each aseptically excised mouse vagina, and epithelial tissue was separated from basement membranes, lamina propria, and muscularis. Tissues were placed in 1 mg/mL Dispase^®^ II solution (Millipore Sigma, Inc., Burlington, MA, USA) containing 0.425 mg/mL collagenase IV and 30 μg/mL DNase I in RPMI w/o methyl red (Thermo Fisher) and incubated at 37°C for 30 minutes for the first round of dissociation [13]. Tissues were then subjected to a second round of dissociation and the mucosal layer was separated from the lamina propria into dishes, minced into small pieces, and incubated for another 30 minutes at 37°C. Cells were washed twice by centrifugation at 900 x *g* for 2 min using RPMI. Dissociated cells were filtered through 70-μm cell strainers before measuring nanoparticle fluorescence by FACS or imaging. After collection, cells were enumerated and their viabilities were estimated with a Cellometer Mini cell counter (Nexcelom Bioscience LLC, Lawrence, MA, USA). Accumulation of nanoparticles in isolated mouse cells was detected by FACS using a 405 nm excitation wavelength. Cells were stained with fluorescently labelled antibodies to EPCAM (CD326)-FITC, F4/80-PE, or CD11c-APC (Invitrogen) according to the manufacturer’s instructions to detect epithelial cells, macrophages and DC, respectively. The percentages of PLGA-PEG + EPCAM, PLGA-PEG + F4/80 and PLGA-PEG + CD11c double-positive cells were analyzed using a Sony SA3800 Spectral Analyzer (Sony Biotechnology, Inc., San Jose, CA, USA).

A fluorescence imaging plate reader (Cytation 3, BioTek, Winooski, VT, USA) equipped with filter cubes for blue (Ex 365, Em 377–477) nm fluorescence emission was used to detect the fluorescence of nanoparticles in vaginal washings, VEC, and tissue homogenates.

### Measurement of vaginal accumulation and systemic translocation of PLGA and *C*. *albicans*

Mouse spleens, livers, kidneys, lungs, and hearts were aseptically excised and homogenized with an Omni homogenizer (Omni International, Inc., Marietta, GA, USA) in 5 mL sterile distilled water. Homogenates were made with the Omni homogenizer set on high for 3–5 x 15 second bursts, with cooling on ice between bursts. The plate reader function of the Cytation 3 was used to detect the total amount of nanoparticle fluorescence (blue channel) per 100 μL homogenate sample. Diluted homogenates were spotted on Sabouraud’s dextrose agar plates (Thermo Fisher), incubated overnight at 37°C, and *C*. *albicans* colonies were counted. Samples of each homogenate were diluted and used for cellular accumulation of nanoparticles by FACS analysis.

### Real-time quantitative reverse-transcription polymerase-chain-reaction (RT-qPCR) gene expression profiling

Custom RT^2^ Profiler qPCR^®^ array applications from Qiagen (Qiagen, Inc., Fredrick, MD, USA) were used to assess expression of mRNA for the genes listed in S1 Table, which may be involved in the response of VEC to contact with *C*. *albicans* as previously described [1]. Total cellular RNA from VEC monolayers was isolated using RNeasy Protect^®^ total RNA isolation kits (Qiagen, Inc., Valencia, CA, USA). Results of the RT-qPCR array experiments were analyzed according to the manufacturer's instructions to determine key signal transduction pathways and immune system interaction genes involved in the PLGA-PEG and *C*. *albicans* effects on VEC.

### Assessment of cell stress mechanisms that induce programmed cell death

Cytotoxicity and cell death were assessed as increased serum concentrations of lactate dehydrogenase (LDH) with a commercial assay (BioVision, Inc., Milpitas, CA, USA). Activation of cell stress mechanisms was assessed using kits run by FACS with a Sony SH800 Cell Sorter or a Sony SA3800 Spectral cell Analyzer (Sony Biotechnology, Inc., San Jose, CA, USA). To determine apoptosis activation, an Apoptosis, DNA Damage, and Cell Proliferation Kit (BD Biosciences, San Jose, CA, USA) was used to measure PARP cleavage and DNA damage response as phosphorylated histone H2AX (γH2AX) by FACS. Positive control Jurkat cells were incubated 4 hours at 37°C with 2 μM staurosporine to induce apoptosis. Results were detected as the % positive cells in gates surrounding the positive control signals. Depolarization of mitochondrial membrane potential (Δψm) was measured with Molecular Probes™ DiIC_1_(5) dye (Invitrogen), which loses fluorescence intensity at 658 nm when the mitochondria depolarize. Positive control Jurkat cells for Δψm depolarization were incubated at 37°C for 5 minutes with 50 μM carbonyl cyanide 3-chlorophenylhydrazone (CCCP). Results were identified as % positive cells with decreased fluorescence intensity as gated on the positive control signals. Oxidative stress that can induce apoptosis was measured as total reactive oxygen species (ROS) by FACS with CellROX^®^ Deep Red™ Reagent according to the manufacturer’s instructions (Invitrogen). Positive control Jurkat cells were induced with 200 μM tert-butyl hydroperoxide incubated at 37°C for 1 hour. Results from samples were identified as fluorescence intensities within gates surrounding the positive control signals. Endoplasmic reticulum stress was assessed using assays for autophagy activation and protein aggresome accumulation in the cells. A CYTO-ID^®^ Autophagy Detection Kit (Enzo Life Sciences, Farmingdale, NY, USA) which employs a 488 nm-excitable green-emitting fluorescent probe to highlight the various vacuolar components of the autophagy pathway was used according to the manufacturer’s instructions to detect induction of autophagy. FACS was used to measure autophagy activation, quantified by CYTO-ID^®^ dye binding to autophagy vesicles. Autophagy induction in positive control Jurkat cells was achieved by incubation at 37°C for 18 hours with 500 nM Rapamycin and 10 μM Chloroquine. Sample results were determined as % positive cells within gates surrounding the positive control signals. Aggresome accumulation in VEC was measured with a ProteoStat Aggresome Detection Kit^®^ (Enzo) by FACS. This assay provides an indication of the degree of endoplasmic reticulum stress in the cells after experimental treatments. Positive controls were induced by incubating 18 hours at 37°C with 10 μM MG-132. Sample results were determined as fluorescence intensities within gates surrounding the positive control signals.

### Cytokine ELISA assays

Vaginal washings were acquired with 4 x 50 μL PBS + 1% BSA from each mouse. Enzyme-linked immunosorbent assays (ELISA) were used to quantify IL-1β, IL-6 and TNFα in vaginal washings (RayBiotech, Inc., Norcross, GA, USA) according to the manufacturer’s instructions. Levels of IL-1β, IL-6 and TNFα in the murine sera were measured using a Bio-Plex^®^ analyzer according to the manufacturer’s instructions (Bio-Rad Life Science Research, Hercules, CA, USA). Analysis was performed using Bio-Plex Manager Software^®^ (Bio-Rad).

### Isolation of mouse DC and PMN

DC were isolated from mouse bone marrow [14]. Femurs and tibiae from mice were removed and purified from muscle tissue by rubbing with tissue paper [15]. After removal of muscles and tissue the bones were incubated in 70% alcohol for 5 minutes for disinfection and rinsed with sterile PBS. After cutting the ends of the bones, the marrow was expelled using a syringe containing PBS through a 25 G needle. Marrow was homogenized by vigorous pipetting followed by centrifugation at 300 x *g* for 5 minutes. Cells were suspended in 9 mL sterile water and 10X PBS was added to make them isotonic. After washing them twice with PBS, cells were suspended in R10 medium (RPMI medium 1640, 10% fetal bovine serum, 100 U/mL penicillin, 100 μg/mL streptavidin, 2 mM L-glutamine, 20 mM HEPES, pH adjusted to 7.0) containing 200 U/mL colony-stimulating factor 2 (CSF2) and incubated for 3 days. At 6 and 8 days, media was replaced with fresh R10 medium with CSF2 [15]. At day 10, cells were analyzed to detect MHCII^+^ CD11c^+^ DC using FACS.

PMN were harvested from peritoneal exudates of mice 18 hours after i.p. injection of 100 μL 10% casein in PBS [16]. PMN were purified [17] by density gradient centrifugation on a cushion of Ficoll-Paque Premium 1.084 (GE Healthcare Biosciences, Pittsburgh, PA, USA). Purified PMN were assessed for purity by FACS with anti-GR-1 antibody (Invitrogen). More than 80% cells were GR-1 positive.

### Chemotaxis assay

Vaginal washings from mice treated with intravaginal PLGA-PEG and *C*. *albicans* were assessed for chemotactic activity for DC and PMN [10]. DC and PMN were collected from mice as described above. Vaginal lavage fluid diluted 1:3 in chemotaxis buffer (RPMI + 1% BSA + 2 mM Glutamax + 25 mM HEPES) was co-cultured with isolated BMDC or PMN in chemotaxis buffer using an HTS 96 Transwell^®^ PL 5 μm (Corning Life Science, Oneonta, NY, USA) system for each cell type, according to the manufacturer’s suggestions. Six wells were used for each sample group. An additional 6 wells were used each for negative chemokine controls (100 ng/mL recombinant mouse CCL5) and positive chemokine controls, 100 ng/mL recombinant mouse CXCL2 for PMN and recombinant mouse CCL21 for DC (Biolegend, San Diego, CA, USA). Migration of DC and PMN to the lower chamber was measured after 1 hour of incubation by counting the cells with a Nexcelom Cellometer Mini automated cell counter (Nexcelom).

### Data analysis

One-way analysis of variance tests with Dunnett's Multiple Comparisons tests on log_10_-transformed data were applied using Prism V. 7.04 software (GraphPad Software, San Diego, CA, USA) to analyze results for statistically significant differences between experimental treatments. Comparisons of statistically significant effects between treatments on internal organ nanoparticle and *C*. *albicans* count data were analyzed by two-way analysis of variance and Sidak’s Multiple Comparisons tests. Results from *C*. *albicans* counts from one replicate were removed as outliers after confirmation using the ROUT test in Prism V. 7.04. Statistical significance was defined by a P*<*0.05 [18].

## Results

### *C*. *albicans* infection increased PLGA-PEG association with VEC and macrophages

**Fig 1:**
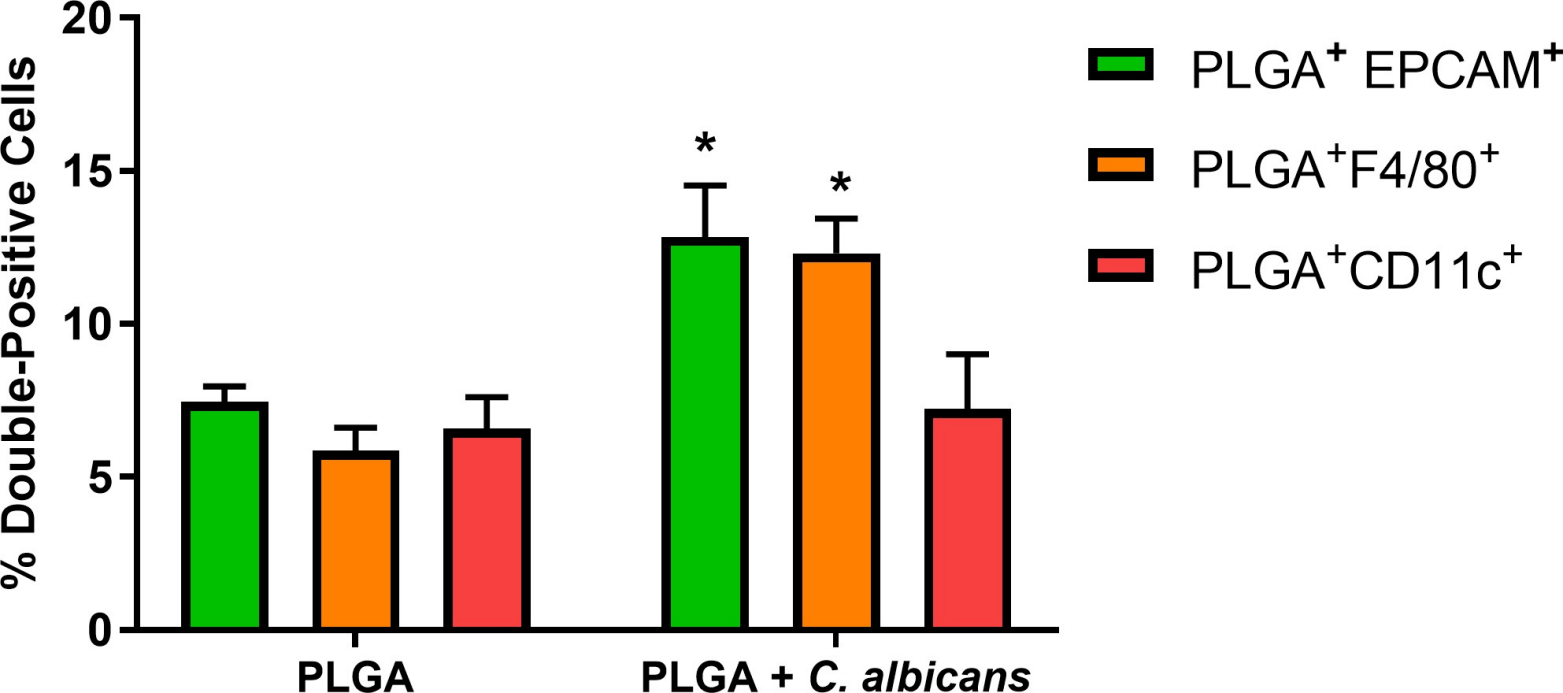
*C*. *albicans* increased co-localization of nanoparticles with cells isolated from vaginal tissues. CBA/J mice were challenged with PLGA-PEG alone, or PLGA-PEG followed by *C*. *albicans*. Cells from the vaginal epithelium were isolated after 18 hours of infection using enzymatic digestion and fluorescent antibody localization of PLGA-PEG with epithelial cells (EPCAM), macrophages (F4/80), and dendritic cells (CD11c) and were analyzed using FACS. Data are Mean ± SEM, n = 6. *Significantly different from the PLGA group, P< 0.05. PLGA-PEG nanoparticles.

PLGA nanoparticles are extensively taken up by eukaryotic cells, macrophages and DC [19]. To study the uptake of drug-delivery nanoparticles by vaginal tissue cells in CBA/J mice, we used fluorescently labelled PLGA-PEG that can be detected by fluorescence imaging and FACS. Cells were stained with fluorochrome-conjugated antibodies to EPCAM, F4/80 or CD11c to detect epithelial cells, macrophages or DC, respectively. FACS was used to measure the percentage of PLGA-PEG^+^EPCAM^+^, PLGA-PEG^+^F4/80^+^, and PLGA-PEG^+^CD11c^+^ double positive cells (Fig 1) 18 hours after challenge with PLGA-PEG or PLGA-PEG and *C*. *albicans*. Significantly more nanoparticles were taken up by PLGA-PEG-treated *C*. *albicans*-infected VEC (2-fold increase) and macrophages (2-fold increase) than PLGA-PEG-treated cells. Association of PLGA-PEG with DC increased 33% by *C*. *albicans* infection, but the results were not statistically significant.

### Vaginal accumulation and systemic translocation of PLGA-PEG and *C*. *albicans*

**Fig 2:**
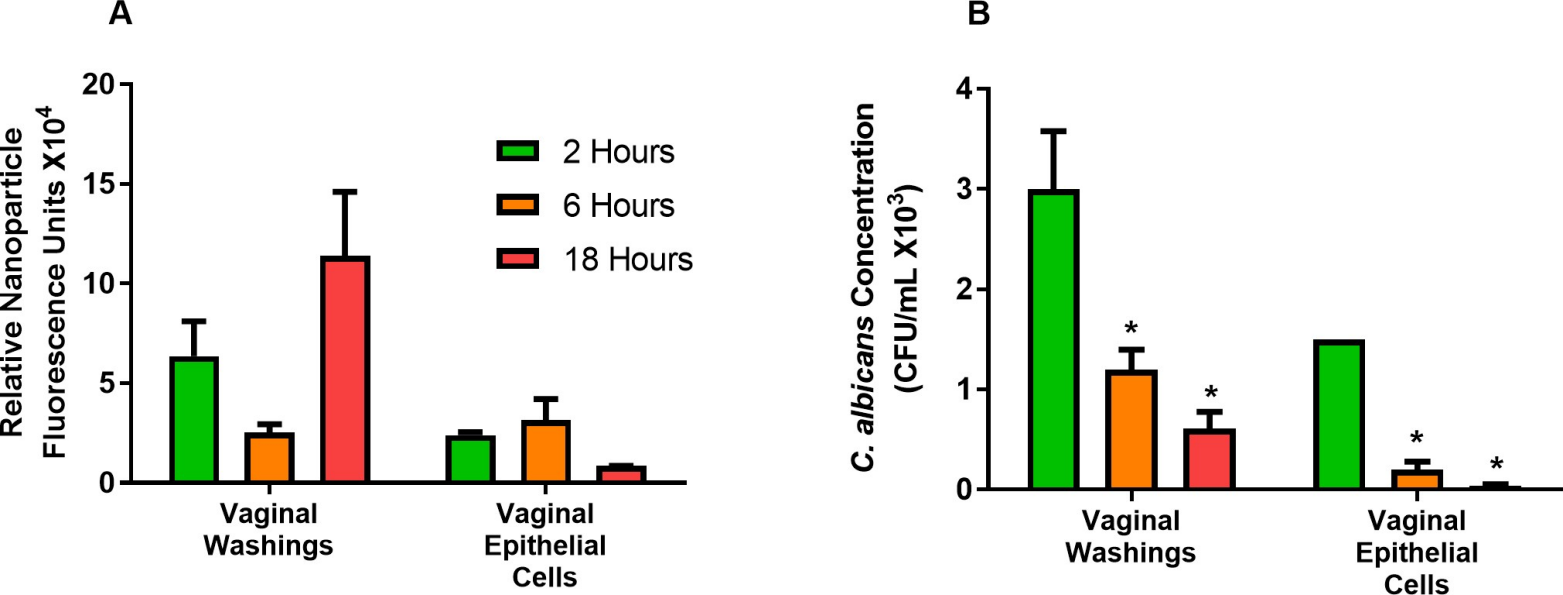
Persistence of nanoparticles and *C*. *albicans* detected in internal organs. Relative fluorescence units of (A) nanoparticles and (B) numbers of viable *C*. *albicans* were measured in vaginal washings and isolated VEC from mice at 2, 6, and 18 hours after *C*. *albicans* challenge. Data are Mean ± SEM, n = 5–6. *Significantly different from the 2-hour group, P< 0.05.

Relative fluorescence of labelled PLGA-PEG and CFU counts of *C*. *albicans* were measured in groups of six mice at 2, 6, and 18 hours after challenge to assess their residence time in the vaginal tract and association with isolated VEC. The PLGA-PEG were observed in vaginal washings and VEC at all three time points. There was a 3-fold decrease in nanoparticle fluorescence between 2 and 18 hours in the VEC, but the change was not statistically significant (Fig 2A). Endocytosis appears to accumulate negatively-charged polymeric nanoparticles in cells at 2 to 6 hours after challenge [20] and exocytosis or degradation may then limit further uptake. In our experiments numbers of *C*. *albicans* isolated from vaginal washings were significantly decreased 3-fold and 4-fold at 6 and 18 hours (Fig 2B). Numbers of *C*. *albicans* isolated from VEC were significantly decreased 7-fold and 37-fold at 6 and 18 hours (Fig 2B). These results show that after the initial dose *C*. *albicans* numbers in the vaginal tract and associated with VEC decreased with time.

**Fig 3:**
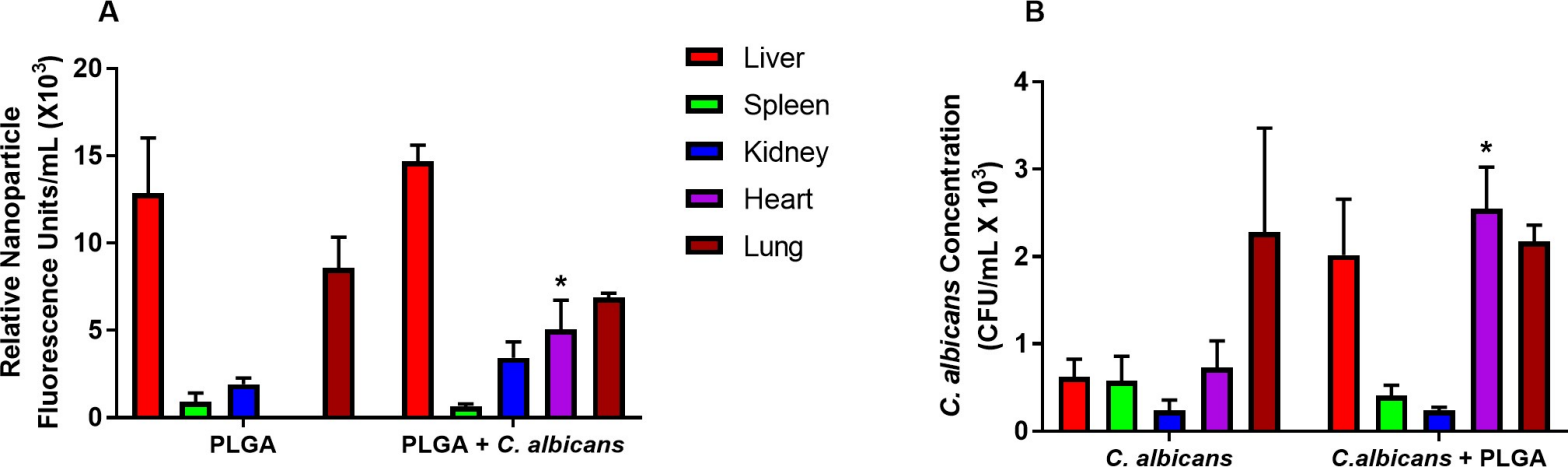
*C*. *albicans* infection increased translocation of PLGA-PEG and yeast to internal organs. (A) Relative fluorescence of PLGA-PEG was compared in livers, spleens, kidneys, hearts, and lungs of mice challenged with PLGA-PEG alone and mice challenged with PLGA-PEG and *C*. *albicans* 18 hours after challenge. (B) *C*. *albicans* colony forming units (CFU) were measured in livers, spleens, kidneys, hearts, and lungs of mice challenged with PLGA-PEG alone and mice challenged with PLGA-PEG and *C*. *albicans* 18 hours after challenge. Data are Mean ± SEM, n = 4–6. *Significantly different from comparison group, P< 0.05. *Ca* = *Candida albicans*; PLGA = PLGA-PEG nanoparticles.

The fluorescence intensity of the PLGA-PEG and *C*. *albicans* CFU in different organs were measured to understand the influence of incubation time on the translocation kinetics of PLGA-PEG and *C*. *albicans*. Accumulation of PLGA-PEG in different organs in the presence or absence of *C*. *albicans* was detected as fluorescence intensity of PLGA-PEG at 18 hours after challenge. The *C*. *albicans* challenge significantly increased the accumulation of PLGA-PEG 5-fold in the heart at 18 hours after challenge (Fig 3A). A significant 3-fold increase in numbers of *C*. *albicans* was also observed in the heart in the presence of PLGA-PEG (Fig 3B), suggesting PLGA-PEG increased systemic distribution of the infection to internal organs after intravaginal PLGA-PEG treatment and *C*. *albicans* infection.

### PLGA-PEG and *C*. *albicans* were cytotoxic to VEC and induced programmed cell death and cellular stress responses

**Fig 4:**
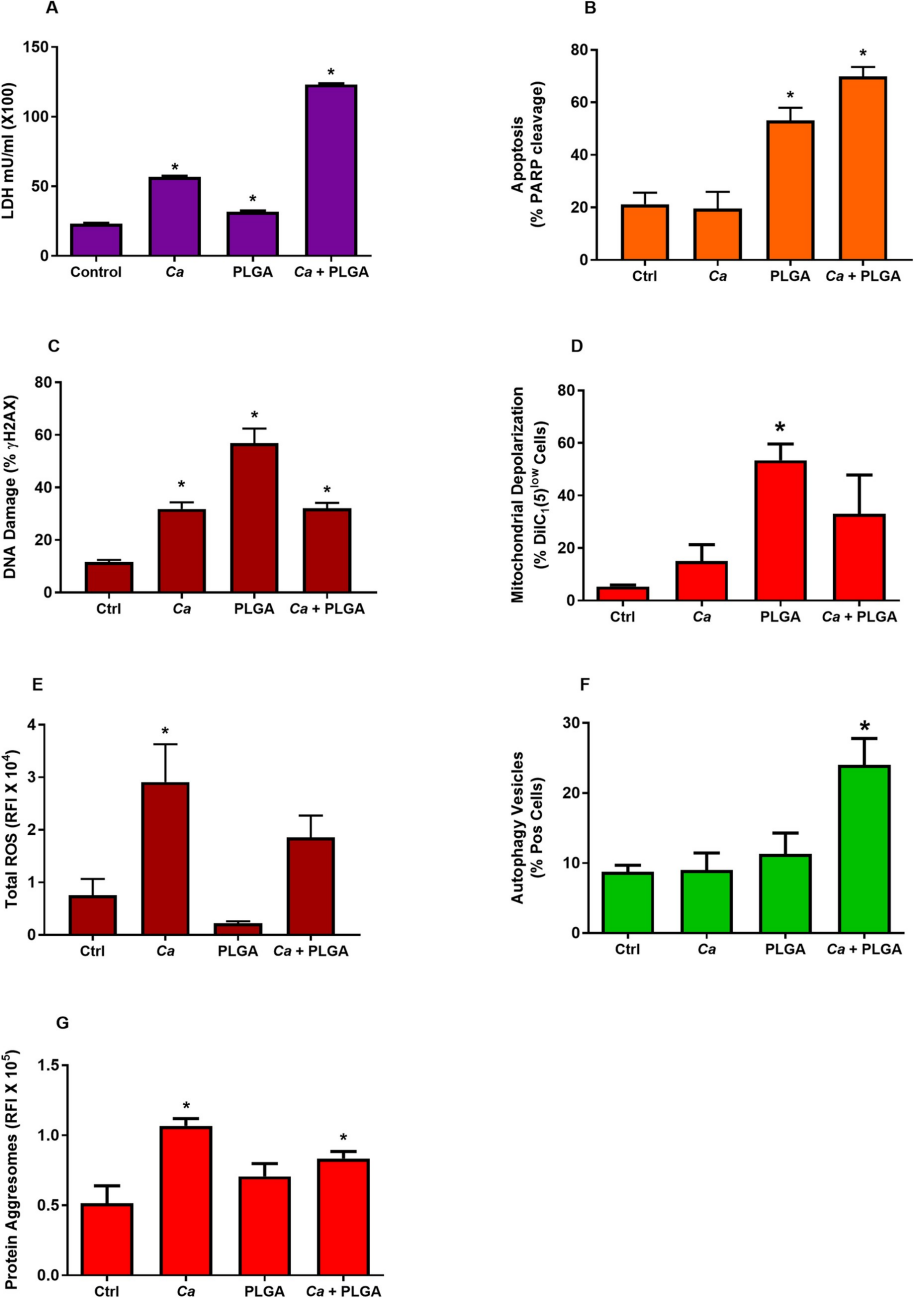
PLGA-PEG induced apoptosis and cell stress responses associated with programmed cell death. (A) Cytotoxicity was measured as elevated concentrations of serum LDH in mice that were infected with *C*. *albicans*, PLGA-PEG-challenged, and PLGA-PEG-challenged *C*. *albicans*-infected compared to untreated control mice. (B) Apoptosis activation was observed as increased cleavage of PARP in VEC of treated mice compared to untreated controls. (C) Cellular responses to DNA damage were compared as the presence of increased γH2AX in VEC compared to VEC of untreated control mice. (D) Depolarization of mitochondrial membrane potential in VEC from treated mice was measured as the percentage of VEC gated for low 658 nm fluorescence from DiIC_1_(5) dye. (E) Total ROS production in VEC was measured as relative fluorescence intensity of CellROX^®^ Deep Red dye in VEC from treated mice. (F) Autophagy was measured as accumulation of Cyto-ID^®^ dye in VEC of treated mice. (G) Protein aggresome accumulation in VEC of treated mice was measured as relative fluorescence intensity of ProteoStat^®^ dye binding. Experimental groups were: untreated controls (Ctrl), *C*. *albicans*-infected only (*Ca*), PLGA-PEG-challenged only (PLGA), and PLGA-PEG-challenged, *C*. *albicans*-infected (PLGA + *Ca*) mice. Data are Mean ± SEM, n = 3–6. *Significantly different from control, P< 0.05.

Cytotoxicity of PLGA-PEG and *C*. *albicans* was detected as increased serum LDH levels in mice at 18 hours after intravaginal challenge. Both the nanoparticles and the fungus caused elevated serum LDH levels compared to the control mice (Fig 4A). Serum LDH levels were significantly elevated 5-fold in PLGA-PEG-treated *C*. *albicans*-infected mice over control mice. *C*. *albicans*-challenged mice and PLGA-PEG-challenged mice showed serum LDH levels 2.5-fold and 1.4-fold higher, respectively, than control mice. Given the significant cytotoxicity of the treatments, it was suspected that activation of programmed cell death would be seen in VEC of the treated mice.

During drug-induced apoptosis PARP is subsequently cleaved into two fragments that contain the active site (89 kDa) and the DNA-binding domain (24 kDa) of the enzyme [21]. This cleavage inactivates the enzyme and it becomes unable to respond to DNA strand breaks. Taking advantage of this property, activation of apoptosis was measured in the present study by detecting cleaved PARP, which accumulates during middle- and late-stages of apoptosis due to Caspase 3 activity. Data showed that the amount of PARP cleavage was significantly increased 2 to 3-fold in VEC isolated from mice after 18 hours stimulation with PLGA-PEG and increased 3 to 4-fold in combination with *C*. *albicans* infection, indicating activation of apoptosis (Fig 4B).

A contributing mechanism that leads to programmed cell death was analyzed by measuring histone H2AX phosphorylation, which increases after cellular DNA damage. Both *C*. *albicans* (2.3-fold increase) and PLGA-PEG (5.4-fold increase) showed significant induction of the DNA damage response, shown as increased γH2AX concentrations in VEC isolated from mice 18 hours after challenge (Fig 4C).

Additional evidence of programmed cell death activation was observed as significant increases in depolarization of mitochondrial membrane potentials by PLGA-PEG, which had 10-fold increased numbers of cells with low dye retention (Fig 4D). *C*. *albicans* infection alone or with PLGA-PEG increased the mitochondrial depolarization 3 to 6-fold, but they were not statistically significant.

A common property of nanoparticles is to cause cellular oxidative stress and the production of ROS [22], which can induce mitochondrial depolarization and subsequent programmed cell death. In the present study, total ROS production was increased 3-fold in both *C*. *albicans*-infected mice and PLGA-PEG-challenged, *C*. *albicans*-infected mice 18 hours after intravaginal challenge (Fig 4E).

Endoplasmic reticulum stress is another mechanism that can activate programmed cell death in VEC treated with PLGA-PEG [1]. In the present study autophagy induction was measured in PLGA-PEG-treated and *C*. *albicans*-infected mice as a marker of the cellular endoplasmic reticulum stress response. One conventional way of monitoring autophagy activity is to measure the increased numbers of autophagosomes in cells responding to autophagic induction. The accumulation of autophagosomes may represent increased generation of autophagosomes or a block in autophagosome maturation. Thus, detection of autophagy vesicles, which includes both autophagosomes and autolysosomes, provides a meaningful way of measuring autophagy [23]. We measured total autophagy vesicle induction in VEC after challenging mice with PLGA-PEG and *C*. *albicans*. FACS data showed 3-fold increased numbers of autophagy vesicles in VEC isolated from PLGA-PEG treated, *C*. *albicans* infected mice compared to the control group (Fig 4F). PLGA-PEG alone also increased autophagy vesicle production 1.4-fold, but the results were not statistically significant. Since autophagy activation in VEC may be related to the unfolded protein response, 2.4-fold increased accumulation of protein aggresomes was measured in the mouse VEC after *C*. *albicans* infection, 1.4-fold after PLGA-PEG treatment, and 1.6-fold in VEC of mice that were both PLGA-PEG-challenged and *C*. *albicans*-infected (Fig 4G). The increase in aggresome accumulation in VEC of PLGA-PEG-challenged mice was not significantly significant, but the other treatments were.

### Production of proinflammatory cytokines was increased by PLGA-PEG and *C*. *albicans*

**Fig 5:**
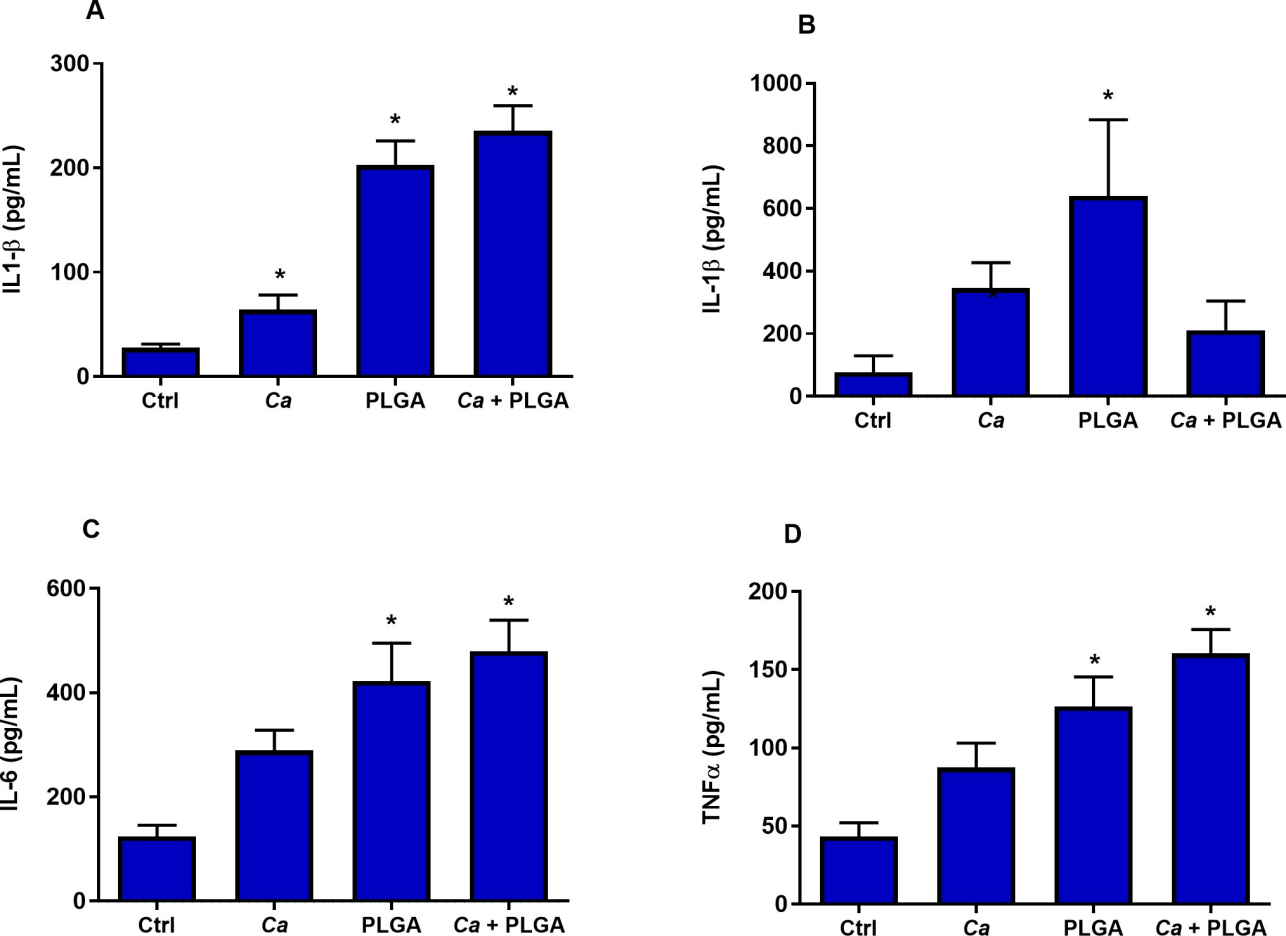
PLGA-PEG increased serum IL-1β, IL-6, and TNFα and IL-1β in vaginal washings. CBA/J mice were challenged with either PLGA-PEG or infected with *C*. *albicans* or treated with PLGA-PEG followed by *C*. *albicans* infection. Blood sera were collected 18 hours post-infection and a Bio-Plex^®^ assay was used to analyze (A) IL-1β, (C) IL-6, and (D) TNFα. (B) ELISA was used to measure IL-1β 18 hours post-infection from vaginal washings. Data represent mean ± SEM, n = 3–6. *Significantly different than control, P<0.05.

Nanoparticles are recognized by the cells of the immune system, and this may lead to immunostimulation, inflammatory responses, or autoimmune disorders [24]. The primary function of the immune system is to protect the host from pathogens and toxins, however, recognition of nanoparticles by the immune cells may also result in uncontrolled immune activation and inflammation. Our in vitro data showed PLGA-PEG induced pro-inflammatory signaling responses in VEC with increased production of cytokines and chemokines, *e*.*g*. IL-1β, TNFα, IL-1α, IL-6, and IL-18 [1]. Secretion of some of these mediators in the mouse sera and vaginal washings was analyzed using Bio-Plex^®^ and ELISA assays. Increased levels of IL-1β, IL-6, and TNFα were detected in sera of PLGA-PEG-treated mice both in the presence and absence of *C*. *albicans*-infection (Fig 5A, 5C and 5D, respectively). These cytokines are important mediators of the inflammatory response and are involved in a variety of cellular activities, e.g. apoptosis and recruitment of inflammatory cells. Increased production of IL-1β was also observed in vaginal washings collected from PLGA-PEG-treated mice compared to control mice (Fig 5B).

### Mouse VEC cell stress and pro-inflammatory mRNA expression were affected by PLGA-PEG and *C*. *albicans*

The effects of *C*. *albicans* challenge, with and without PLGA-PEG, on mRNA expression of 167 genes by an RT-qPCR assay (S1 Table) showed that genes involved in apoptosis activation were suppressed in vaginal tissues of mice infected intravaginally with *C*. *albicans* (Table 1). *C*. *albicans* infection was associated with reduced expression of pro-apoptotic mRNA, *e*.*g*., bcl2/adenovirus e-18, 19-kDa interacting protein 3 (*Bnip3*), apoptosis-related cysteine protease 1 (*Casp1*), and hypoxia-inducible factor 1 alpha (*Hif1a*). *C*. *albicans* infection was also associated with induction of anti-apoptotic B-cell CCL/lymphoma 2 (*Bcl2*), and NLR family apoptosis inhibiting protein (*Naip1*). Expression of mRNA associated with DNA damage that can initiate apoptosis, e.g., ATM serine threonine kinase (*Atm*) and C-reactive protein (*Crp*) were induced by *C*. *albicans*, but *Atm* was not induced by PLGA-PEG.

**Table 1:** Changes in expression of mRNA from cell stress and inflammatory response genes.

Gene Name	RefSeq ID	*C*. *albicans*	PLGA	*C*. *albicans* + PLGA-PEG
		Fold Change in Expression		
*Bcl2*	NM_009741	+8.6	+2.7	+1.7
*Bnip3*	NM_009760	-7.8	-1.5	+1.0
*Casp1*	NM_009807	-6.0	-2.2	-1.4
*Ccna2*	NM_009828	-4.2	-1.9	-1.4
*Cdkn1b*	NM_009875	-3.1	+1.3	-1.1
*Egfr*	NM_007192	-6.8	-1.3	-1.5
*Hif1a*	NM_010431	-6.1	-1.3	-1.1
*Naip 1*	NM_008670	+2.6	+3.1	+1.2
*S100a8*	NM_013650	-4.6	-1.9	-2.0
*Atg3*	NM_026402	-5.6	-1.8	-1.3
*Atm*	NM_007499	+5.2	-1.4	-1.3
*Calr*	NM_007591	-4.2	+1.1	+1.1
*Cbbl1*	NM_134048	+5.6	-1.4	-1.1
*Cct4*	NM_009837	-3.3	-1.4	-1.2
*CRP*	NM_007768	+27.7	+4.7	+3.3
*Ganc*	NM_172672	+3.4	+1.2	-1.1
*Krt8*	NM_031170	+6.2	+7.6	+1.1
*Krt18*	NM_010664	+2.3	+3.0	-1.1
*Ager*	NM_007425	+38.3	-1.3	-1.3
*Ccl5*	NM_013653	+4.0	+1.7	+1.3
*Cd1d1*	NM_007639	+3.7	-1.3	+1.1
*Cd36*	NM_007643	+3.1	+3.1	+2.5
*Cd40*	NM_011611	+4.1	-1.4	-1.3
*Cd300lf*	NM_145634	+3.9	+1.0	+1.0
*Cxcl1*	NM_008176	+6.4	-1.6	-1.3
*Il1a*	NM_010554	-4.3	-2.3	-2.0
*Il6*	NM_031168	+1.0	-6.8	-2.3
*Il23a*	NM_031252	+3.0	-2.2	+1.3
*Irf7*	NM_016850	+3.1	+2.2	-1.0
*Itgb2*	NM_008404	+3.7	+1.6	+1.1
*Nos2*	NM_010927	-1.4	+6.5	+1.4
*Prtn3*	NM_011178	+2.1	+8.1	+2.6

Changes less than ± 2-fold are considered no effect on expression. Data represent the average of 4 samples/treatment: *P<0.05.

Stress on endoplasmic reticulum function, protein traffic, and autophagy was observed as changes in expression of associated genes in the vaginal tissues of mice challenged with *C*. *albicans* and with PLGA-PEG. *C*. *albicans* infection was associated with reduced expression of mRNA associated with autophagy, e.g., autophagy-related gene protein 3 (*Atg3*), and ER stress, e.g., calreticulin (*Calr*) and chaperonin-containing TCP1 subunit 4 (*Cct4*), which were not affected by PLGA-PEG. Some inflammation-associated mRNA were induced by *C*. *albicans*, e.g., advanced glycosylation end-product specific receptor (*Ager*), and C-X-C motif chemokine ligand 1 (*Cxcl1*), while others were induced by PLGA-PEG, e.g., nitric oxide synthase 2 (*Nos2*), and proteinase 3 (*Prtn3*). Some of the mRNA induced by *C*. *albicans* were suppressed by PLGA-PEG, *e*.*g*., *Ager*, C-C motif chemokine ligand 5 (*Ccl5*), *Cd1d1*, *Cxcl1*, and integrin subunit beta 2 (*Itgb2*). The PLGA-PEG did not significantly induce apoptosis activation-associated mRNA, but it did induce expression of *Bcl2* and *Naip1* mRNA.

### BMDC and PMN chemotaxis is induced by *C*. *albicans* and PLGA-PEG

**Fig 6:**
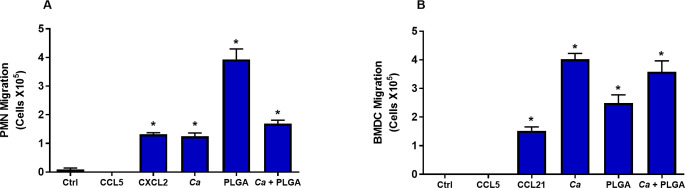
Vaginal washings collected from control, *C*. *albicans-*infected, PLGA-PEG-treated and PLGA-PEG-treated, *C*. *albicans-*infected mice significantly promoted chemotaxis of PMN and BMDC. (A) PMN and (B) DC chemotaxis was measured as numbers of cells that migrated across a Transwell™ PL 5 nm membrane system. CCL5 was a negative migration control. CXCL2 and CCL21 were positive migration controls for PMN and DC, respectively. Data are Mean ± SEM, n = 5–6. *Significantly different from control, P<0.05. *Ca* = *Candida albicans*; PLGA = PLGA-PEG nanoparticles.

Leukocyte recruitment via chemotaxis is an important component of the inflammatory response. Circulating leukocytes in the bloodstream migrate towards the site of inflammation in response to a complex network of proinflammatory signalling molecules including cytokines, chemokines, prostaglandins and microbial-derived chemoattractants that are produced either endogenously or exogenously. Migration of DC and other immune cells such as PMN to sites of infection and inflammation is a critical step toward an effective defense against pathogens, but it is also considered a sign of inflammation [25]. Our data showed vaginal washings collected from *C*. *albicans*-infected, PLGA-PEG-treated, and PLGA-PEG treated *C*. *albicans*-infected mice and the positive control (CCL21) significantly promoted DC recruitment across a Transwell^®^ PL 5 system and showed enhanced DC migration in the chemotaxis assay. No migration was observed when DC were co-cultured with the negative control (CCL5) and vaginal washings collected from control mice (Fig 6). A significantly greater level of PMN migration across the Transwell^®^ was observed when they were co-cultured with vaginal washings collected from positive control (CXCL2), *C*. *albicans*-infected, PLGA-PEG–treated, and PLGA-PEG-treated, *C*. *albicans*-infected mice compared to the controls; however, no migration was observed when PMN were co-cultured with negative control (CCL5) and vaginal washings collected from control mice (Fig 6). These data revealed a crucial role of PLGA-PEG in the induction of BMDC and PMN chemotaxis and provide additional support for the emerging concept of PLGA-PEG as a potential cause of inflammation.

## Discussion

The PLGA polymer is one of the most widely used biodegradable polymers in medical applications, with a proven record of safety [26]. Recent studies highlighted the use of PLGA nanoparticles as a potential drug delivery system to carry different types of highly potent and fragile drugs, proteins and various other macromolecules such as DNA, RNA and peptides [27]. Its ability to deliver different types of cargo efficiently and compatibly with other polymers makes PLGA an attractive choice as a drug carrier. PLGA polymer is most popular among the various available biodegradable polymers because of its long clinical experience, favorable degradation characteristics and use for sustained drug delivery. Transport of therapeutic agents to the vaginal epithelium is limited by barriers such as fluid flow, pH, mucous, and epithelial cell tight junctions [2]. Polymeric nanoparticles can provide controlled release of drugs, but must have surfaces modified, *e*.*g*., with PEG, to decrease adherence to mucous that can wash them away from the vaginal mucosa [28]. In the present study PEG-modified PLGA nanoparticles were evaluated for their ability to reach the vaginal epithelium and to observe tissue responses in a mouse model.

Colocalization of PLGA-PEG with VEC was observed as rapid uptake by VEC (2 hours) and retention up to 18 hours in the in vivo model. A previous study using cultures of DC and macrophages isolated from peripheral blood leukocytes showed that after 24 hours incubation these cells phagocytosed PLGA nanospheres [19]. These in vitro data demonstrated that DC can take up PLGA as efficiently as macrophages. In the present study we showed that not only DC and macrophages, but also VEC take up PLGA-PEG in vivo and are also capable of inducing cytotoxic and pro-inflammatory responses.

In our previous study of PLGA-PEG treatment and *C*. *albicans* infection of the human VK2(E6/E7) VEC cell line, cytotoxic mechanisms were detected that included oxidative stress, DNA damage repair, ER stress, and mitochondrial membrane depolarization, which led to apoptosis [1]. Apoptosis is a form of programmed cell death where mitochondria play a key role [29]. Mitochondria regulate apoptosis at several levels, for example maintenance of ATP production [30], mitochondrial membrane potential (Δψ_m_) [31], and mitochondrial membrane permeability [32]. A relevant example is the observation that silica nanoparticles cause oxidative stress and loss of mitochondrial membrane potential in glioblastoma cells [33]. In the present study we observed cellular apoptosis in murine VEC after stimulation with PLGA-PEG that was not associated with oxidative stress, but there was significant response to DNA damage and mitochondrial depolarization. Further support of the role of apoptosis influenced by PLGA-PEG in the current in vivo study was the observation of increased PARP cleavage in VEC isolated from *C*. *albicans* infection, PLGA-PEG-treatment, and in PLGA-PEG-treated, *C*. *albicans*-infected mice, which correlated with our previous in vitro experiments.

Cellular uptake of nanoparticles by VEC may stress endocytic trafficking activating autophagy and unfolded protein response mechanisms in the endoplasmic reticulum. This activity leads to ER stress that can induce apoptosis by the canonical pathway that involves mitochondria. Our previous in vitro study showed PLGA-PEG nanoparticles induced expression of autophagy-associated genes, increased autophagy vesicle production, and accumulation of protein aggresomes in the ER of human VEC [1], but unlike the previous in vitro study, our mouse study showed induction of autophagy vesicle production, but not increased autophagy-associated gene expression in VEC from PLGA-PEG-treated *C*. *albicans*-infected mice. While protein aggresome accumulation was observed in *C*. *albicans*-infected and PLGA-PEG-treated *C*. *albicans*-infected mice, unaffected expression of *Calr* and *Cct4* mRNA in VEC from the mice was also observed, suggesting that ER stress was not the only driving factor for VEC apoptosis in vivo. Due to the significant induction of mitochondrial depolarization in the VEC collected from PLGA-PEG-treated and PLGA-PEG-treated *C*. *albicans*-infected mice, we suggest that PLGA-PEG induced apoptosis in VEC through a mitochondrial depolarization-associated pathway initiated by damage to intracellular transport mechanisms. Excessive activation of autophagy can induce necroptosis through the RIPK1 pathway or apoptosis by a Na^+^, K^+^-ATPase-mediated pathway [34]. We did not observe increased expression of *Ripk1* or *Ripk2* mRNA in mice treated with PLGA-PEG or *C*. *albicans*, so necroptosis is not a likely contributor to the VEC cell death we observed.

Another contributor to VEC cell death can be the process of pyroptosis, which involves activation of NLRP3 inflammasomes, which cause secretion of pro-inflammatory cytokine IL1B. It is known that nanoparticles can induce pyroptosis, for example, silver nanoparticles induced inflammasome formation and production of IL1B upon incubation with human monocytes [35]. In the present study, induction of inflammasomes was inferred from increased levels of IL-1β in the sera and vaginal washings of *C*. *albicans*-infected mice and PLGA-PEG-treated mice. Pyroptosis and the resulting inflammation is characteristic of vaginal candidiasis and was observed in our in vitro study with a human VEC cell line [1]. In the present study pyroptosis can be inferred as the cause of vaginal inflammation in mice. The proinflammatory cytokines IL-1β, IL-6 and TNFα are often analyzed to evaluate immunomodulatory effects and immunotoxicity of nanomaterials [36]. Our data also showed increased levels of IL-6 and TNFα in the blood serum collected from PLGA-PEG-treated and PLGA-PEG-treated, *C*. *albicans*-infected mice. The presence of these cytokines in the blood sera of PLGA-PEG-treated and PLGA-PEG-treated, *C*. *albicans*-infected mice provides further evidence of immunotoxicity associated with PLGA-PEG and how it exacerbates *C*. *albicans*-associated inflammation. In further support of our findings, a previous study showed that cellular uptake of PLGA by J774 macrophages induced activation of NF-κB signaling followed by production of inflammatory cytokines TNFα and IL-1β [37].

Besides the elevated concentrations of pyroptosis-related and pro-inflammatory cytokines in the sera and vaginal washings of PLGA-PEG-treated mice, *C*. *albicans*-infected mice, and PLGA-PEG-treated, *C*. *albicans*-infected mice, expression of inflammation-associated gene mRNA were increased in this study. More specifically, we measured local inflammation in the vaginal tract by co-culturing the vaginal lavage fluids from the mice treated with PLGA-PEG and *C*. *albicans* with DC and PMN in a chemotaxis assay. DC and PMN chemotaxis were induced by both *C*. *albicans* infection and PLGA-PEG treatment. DC are among the first cells to recognize invading pathogens and initiate an immune response. This initial innate response may dictate the subsequent type of adaptive immune response. The ability of DC to produce cytokines and to induce expression of HLA class I/II and co-stimulatory markers and the potential to produce critical cytokines makes them the primary and most efficient activators of adaptive immunity [38]. Maturation of DC by PLGA stimulation has been shown in several studies. In one example human peripheral blood monocyte-derived immature dendritic cells co-cultured with PLGA resulted in DC maturation [39]. A recent study showed PLGA exposure causes expression of maturation markers and cytokine release in primary DC cultures [40]. Recruitment of DC is considered a hallmark of an acute inflammatory response at mucosal surfaces [41]. Our in vivo study also demonstrated induction of chemotaxis of DC and PMN, suggesting induction of inflammation by PLGA-PEG and *C*. *albicans* infection. PMN act as the predominant phagocytic cells for first-line defense to be recruited to an inflammatory site against infection or xenobiotics and the toxicity of nanoparticles can be mediated by the release of factors from PMN [42].

Gene expression analysis results reflected the pro-inflammatory, anti-apoptotic effects we have previously observed with *C*. *albicans* infections of human VEC. They also showed that PLGA treatment in vivo is less toxic to murine VEC than we previously observed in human VEC in vitro. The difference between the two studies most likely reflects the longer exposure time of a continuous dose of nanoparticles to VEC in vitro versus the shorter exposure of a single application to the mucosal surface in vivo. Results suggest that manipulations to the design of the nanoparticle surface presentation of PEG may further reduce cytotoxicity. In support of this, studies have shown that PLGA nanoparticles without PEG on their surface have low cytotoxicity [26].

In recent years nanoparticle-based drug delivery platforms have received attention for vaginal drug delivery, since nanoparticles can provide sustained release, cellular targeting, and intrinsic adjuvant properties of microbicidal drugs that are necessary for maintaining protective drug concentrations and to improve the efficacy of these drugs. This is because drug release from nanoparticles can result in more controlled vaginal absorption compared to a vaginal gel, although careful attention to formulations are essential to maximize the benefits and minimize potential problems [43]. The current study clarifies how the host inflammatory response to the PLGA-PEG adds to the host inflammatory response to yeast by showing induction of autophagy, apoptosis, proinflammatory cytokine production and chemotaxis of DC and PMN to the inflammation site. Further study is needed to understand the interaction of PLGA-PEG with the VEC receptors as a mechanism of cellular entry and modulation of cell signaling pathways for induction of inflammation. Detailed understanding of the mechanisms shown in this study will help future researchers and drug manufacturers to improve the design of PLGA-PEG drug delivery vehicles to be more efficient and cause less inflammation.

## Conclusions

In a mouse model of vaginal candidiasis, PLGA-PEG nanoparticles induced cell stress and apoptosis in vaginal epithelial cells and promoted a strong inflammatory response in addition to the inflammatory response to *C*. *albicans* infection. The nanoparticles were associated with increased translocation of the fungus from the vaginal tract to internal organs and the *C*. *albicans* infection increased translocation and persistence of the nanoparticles in internal organs. These observations suggest that intravaginal drug-delivery with mucous-penetrating PLGA-PEG nanoparticles might aggravate the morbidity of active vaginal yeast infections in women.

## Supporting information

S1 Table Changes in expression of mRNA from 166 cell stress and inflammatory response genes, including genes that were unaffected by the treatments. (Data represent the average of four samples/treatment: *P<0.05).
(DOCX)

S1 File Data subjected to analysis to build graphs and statistical comparisons reported in this manuscript. (DOCX)

S2 File Histograms and dot plots showing gates used to measure RFI and % positive cells results relative to the positive controls. (PDF)

## References

[1] Wagner RD, Johnson SJ, Danielsen ZY, Lim JH, Mudalige T, Linder S. Polyethylene glycol-functionalized poly (Lactic Acid-co-Glycolic Acid) and graphene oxide nanoparticles induce pro-inflammatory and apoptotic responses in *Candida albicans*-infected vaginal epithelial cells. PLoS One. 2017 4 3;12 (4): e0175250 10.1371/journal.pone.0175250 28369145 PMC5378405

[2] Rosati P, Bruno M, Jaeger M, tenOver J, Netea MG. Recurrent vulvovaginal candidiasis: an immunological perspective. Microorganisms. 2020 8;8: 144–157. 10.3390/microorganisms8020144 PMC7074770 31972980

[3] Fidel PL Jr. Immunity in vaginal candidiasis. Curr Opin Infect Dis. 2005;18: 107–111. 10.1097/01.qco.0000160897.74492.a3 15735412

[4] Wong TW, Dhanawat M, Rathbone MJ. Vaginal drug delivery: strategies and concerns in polymeric nanoparticle development. Expert Opin Drug Deliv. 2014;11(9): 1419–1434. 10.1517/17425247.2014.924499 24960192

[5] Whaley KJ, Hanes J, Shattock R, Cone RA, Friend DR. Novel approaches to vaginal delivery and safety of microbicides: biopharmaceuticals, nanoparticles, and vaccines. Antiviral Res. 2010;885: 555–556. 10.1016/j.antiviral.2010.09.006 21109069

[6] Zhang T, Sturgis TF, Youan BC. pH-responsive nanoparticles releasing tenofovir intended for the prevention of HIV transmission. Eur J Pharm Biopharm. 2011;79: 526–536. 10.1016/j.ejpb.2011.06.007 21736940 PMC3375322

[7] Li JJ, Hartono D, Ong CN, Bay BH, Yung LY. Autophagy and oxidative stress associated with gold nanoparticles. Biomaterials. 2010;31: 5996–6003. 10.1016/j.biomaterials.2010.04.014 20466420

[8] Yu L, Lu Y, Man N, Yu SH, Wen LP. Rare earth oxide nanocrystals induce autophagy in HeLa cells. Small. 2009;5: 2784–2787. 10.1002/smll.200901714 19885892

[9] Li H, Li Y, Jiao J, Hu HM. Alpha-alumina nanoparticles induce efficient autophagy-dependent cross-presentation and potent antitumor response. Nat Nanotechnol. 2011;6: 645–650. 10.1038/nnano.2011.153 21926980 PMC3483867

[10] Yano J, Lilly E, Barousse M, Fidel PL Jr. Epithelial cell-derived S100 calcium-binding proteins as key mediators in the hallmark acute neutrophil response during Candida vaginitis. Infect Immun. 2010;78: 5126–5137. 10.1128/IAI.00388-10 20823201 PMC2981313

[11] Rahman D, Mistry M, Thavaraj S, Naglik JR, Challacombe SJ. Murine model of concurrent oral and vaginal *Candida albicans* colonization. Meth Mol Biol. 2012;845: 527–535. 10.1007/978-1-61779-539-8_38 22328401

[12] Wagner RD, Johnson SJ. Probiotic Lactobacillus and estrogen effects on vaginal epithelial gene expression responses to *Candida albicans*. J Biomed Sci. 2012;19: 58–66. 10.1186/1423-0127-19-58 22715972 PMC3404894

[13] Iijima N, Linehan MM, Saaland S, Iwasaki A. Vaginal epithelial dendritic cells renew from bone marrow precursors. Proc Natl Acad Sci USA. 2007;104(48): 19061–19066. 10.1073/pnas.0707179104 18006657 PMC2141908

[14] Kent D, Dykstra B, Eaves C. Isolation and assessment of long-term reconstitution hematopoietic stem cells from adult mouse bone marrow. Curr Protoc Stem Cell Biol. 2007;3: 2A.4.1–2A.4.23. 10.1002/9780470151808.sc02a04s3 18785176

[15] Lutz MB, Kukutsch N, Ogilvie AL, Rossner S, Koch F, Romani N, et al An advanced culture method for generating large quantities of highly pure dendritic cells from mouse bone marrow. J Immunol Methods. 1999;223: 77–92. 10.1016/s0022-1759(98)00204-x 10037236

[16] Swamydas M, Luo Y, Dorf ME, Lionakis MS. Isolation of Mouse Neutrophils. Curr Protoc Immunol. 2015;110: 3.20.1–3.20.15. 26237011 10.1002/0471142735.im0320s110 PMC4574512

[17] Phelan K, May KM. Basic techniques in mammalian cell tissue culture. Curr Protoc Cell Biol. 2015;66: 1.1.1–1.1.22. 10.1002/0471143030.cb0101s66 25727327

[18] Motulsky H. Intuitive biostatistics. New York, NY: Oxford University Press, 1995.

[19] Lutsiak ME, Robinson DR, Coester C, Kwon GS, Samuel J. Analysis of poly(D,L-lactic-co-glycolic acid) nanosphere uptake by human dendritic cells and macrophages *in vitro*. Pharm Res. 2002;19: 1480–1487. 10.1023/a:1020452531828 12425465

[20] Oh N, Park J-H. Endocytosis and exocytosis of nanoparticles in mammalian cells. Int J Nanomed. 2014;9(Suppl 1): 51–63. 10.2147/IJN.S26592 PMC4024976 24872703

[21] Kaufmann SH, Desnoyers S, Ottaviano Y, Davidson NE, Poirier GG. Specific proteolytic cleavage of poly(ADP-ribose) polymerase: an early marker of chemotherapy-induced apoptosis. Cancer Res. 1993;53: 3976–3985. 8358726

[22] Manke A, Wang L, Rojanasakul Y. Mechanisms of nanoparticle-induced oxidative stress and toxicity. Biomed Res Int. 2013 8 20; e942916 10.1155/2013/942916 24027766 PMC3762079

[23] Mizushima N, Yoshimori T, Levine B. Methods in mammalian autophagy research. Cell. 2010;140: 313–326. 10.1016/j.cell.2010.01.028 20144757 PMC2852113

[24] Zolnik BS, Gonzalez-Fernandez A, Sadrieh N, Dobrovolskaia MA. Nanoparticles and the immune system. Endocrinol. 2010;151: 458–465. 10.1210/en.2009-1082 PMC2817614 20016026

[25] Selders GS, Fetz AE, Radic MZ, Bowlin GL. An overview of the role of neutrophils in innate immunity, inflammation and host-biomaterial integration. Regen Biomaterials. 2017;4: 55–68. 10.1093/rb/rbw041 PMC5274707 28149530

[26] Machado A, Cunha-Reis C, Araújo F, Nunes R, Seabra V, Ferreira D, et al Development and *in vivo* safety assessment of tenofovir-loaded nanoparticles-in-film as a novel microbicide delivery system. Acta Biomater. 2016;44: 332–340. 10.1016/j.actbio.2016.08.018 27544812

[27] Liang GF, Zhu YL, Sun B, Hu FH, Tian T, Li SC, et al PLGA-based gene delivering nanoparticle enhance suppression effect of miRNA in HePG2 cells. Nanoscale Res Lett. 2011;6: 447 10.1186/1556-276X-6-447 21749688 PMC3211866

[28] Cu Y, Booth CJ, Saltzman WM. *In vivo* distribution of surface-modified PLGA nanoparticles following intravaginal delivery. J Control Release. 2011;156: 258–264. 10.1016/j.jconrel.2011.06.036 21763739 PMC3220785

[29] Desagher S, Martinou JC. Mitochondria as the central control point of apoptosis. Trends Cell Biol. 2000;10: 369–377. 10.1016/s0962-8924(00)01803-1 10932094

[30] Leist M, Nicotera P. The shape of cell death. Biochem Biophys Res Commun. 1997;236: 1–9. 10.1006/bbrc.1997.6890 9223415

[31] Zoratti M, Szabo I. The mitochondrial permeability transition. Biochim Biophys Acta. 1995;1241: 139–176. 10.1016/0304-4157(95)00003-a 7640294

[32] Kroemer G, Reed JC. Mitochondrial control of cell death. Nat Med. 2000;6: 513–519. 10.1038/74994 10802706

[33] Kretowski R, Kusaczuk M, Naumowicz M, Kotynska J, Szynaka B, Cechowska-Pasko M. The effects of silica nanoparticles on apoptosis and autophagy of glioblastoma cell lines. Nanomaterials (Basel). 2017;7: 230–252. 10.3390/nano7080230 PMC5575712 28825685

[34] Matsuzawa-Ishimoto Y, Hwang S, Cadwell K. Autophagy and inflammation. An Rev Immunol. 2018;36: 73–101. 10.1146/annurev-immunol-042617-053253 29144836

[35] Yang EJ, Kim S, Kim JS, Choi IH. Inflammasome formation and IL-1beta release by human blood monocytes in response to silver nanoparticles. Biomaterials. 2012;33: 6858–6867. 10.1016/j.biomaterials.2012.06.016 22770526

[36] Elsabahy M, Wooley KL. Cytokines as biomarkers of nanoparticle immunotoxicity. Chem Soc Rev. 2013;42: 5552–5576. 10.1039/c3cs60064e 23549679 PMC3665642

[37] Nicolete R, dos Santos DF, Faccioli LH. The uptake of PLGA micro or nanoparticles by macrophages provokes distinct in vitro inflammatory response. Int Immunopharmacol. 2011;11: 1557–1563. 10.1016/j.intimp.2011.05.014 21621649

[38] Iwasaki A, Medzhitov R. Regulation of adaptive immunity by the innate immune system. Science. 2010;327: 291–295. 10.1126/science.1183021 20075244 PMC3645875

[39] Yoshida M, Babensee JE. Poly(lactic-co-glycolic acid) enhances maturation of human monocyte-derived dendritic cells. J Biomed Mater Res A. 2004;71: 45–54. 10.1002/jbm.a.30131 15368253

[40] Barillet S, Fattal E, Mura S, Tsapis N, Pallardy M, Hillaireau H, et al Immunotoxicity of poly (lactic-co-glycolic acid) nanoparticles: influence of surface properties on dendritic cell activation. Nanotoxicology. 2019;13: 606–622. 10.1080/17435390.2018.1564078 30760076

[41] McWilliam AS, Nelson D, Thomas JA, Holt PG. Rapid dendritic cell recruitment is a hallmark of the acute inflammatory response at mucosal surfaces. J Exp Med. 1994;179: 1331–1336. 10.1084/jem.179.4.1331 8145044 PMC2191461

[42] Lin MH, Lin CF, Yang SC, Hung CF, Fang JY. The Interplay Between Nanoparticles and Neutrophils. J Biomed Nanotechnol. 2018;14: 66–85. 10.1166/jbn.2018.2459 29463366

[43] Cook MT, Brown MB. Polymeric gels for intravaginal drug delivery. J Control Release. 2018;270: 145–157. 10.1016/j.jconrel.2017.12.004 29223536

