# Supplementary material for: Intravaginal poly-(D, L-lactic-co-glycolic acid)-(polyethylene glycol) drug-delivery nanoparticles induce pro-inflammatory responses with Candida albicans infection in a mouse model
Source: PLoS One. 2020 Oct 22;15(10):e0240789. doi: 10.1371/journal.pone.0240789 (PMC7580924; doi:10.1371/journal.pone.0240789)
Supplement: S1 Table — (Data represent the average of four samples/treatment: *P<0.05). (DOCX) [file pone.0240789.s001.docx]

S1 Table. Fold change in expression of genes tested by RT-qPCR

|  |  | **(Fold Change in Expression)** | | |
| --- | --- | --- | --- | --- |
|  |  |  | | |
| **Gene Name** | **RefSeq ID** | ***C. albicans*** | **PLGA-PEG** | ***C. albicans* + PLGA-PEG** |
| *Acvr1* | NM_007394 | +2.3 | +1.0 | +1.2 |
| *Ager* | NM_007425 | +38.3 | -1.3 | -1.3 |
| *Akt1* | NM_009652 | +1.7 | +1.1 | -1.1 |
| *Amfr* | NM_011787 | +1.4 | -1.2 | -1.2 |
| *Anxa1* | NM_010730 | -1.3 | -2.2 | -2.1 |
| *Apaf1* | NM_009684 | +1.3 | +1.2 | -1.1 |
| *Arf6* | NM_007481 | -1.1 | -2.1 | -1.3 |
| *Arrb2* | NM_145429 | +3.1 | -1.8 | +1.2 |
| *Atg3* | NM_026402 | -5.6 | -1.8 | -1.3 |
| *Atg5* | NM_053069 | +1.0 | -1.2 | -1.2 |
| *Atm* | NM_007499 | +5.2 | -1.4 | -1.3 |
| *Atr* | NM_019864 | -1.9 | -1.3 | -1.1 |
| *Bcl2* | NM_009741 | +8.6 | +2.7 | +1.7 |
| *Bid* | NM_007544 | -1.6 | -1.2 | +1.0 |
| *Bmf* | NM_138313 | +2.4 | +1.2 | -1.2 |
| *Bnip3* | NM_009760 | -7.8 | -1.5 | +1.0 |
| *Cab39l* | NM_026908 | +1.4 | -1.4 | -1.2 |
| *Calr* | NM_007591 | -4.2 | +1.1 | +1.1 |
| *Casp1* | NM_009807 | -6.0 | -2.2 | -1.4 |
| *Casp3* | NM_009810 | -1.8 | -1.1 | -1.1 |
| *Cbbl1* | NM_134048 | +5.6 | -1.4 | -1.1 |
| *Ccl5* | NM_013653 | +4.0 | +1.7 | +1.3 |
| *Ccna2* | NM_009828 | -4.2 | -1.9 | -1.4 |
| *Ccnf* | NM_007634 | +2.9 | +1.2 | +1.2 |
| *Cct3* | NM_009836 | +1.2 | -1.1 | +1.1 |
| *Cct4* | NM_009837 | -3.3 | -1.4 | -1.2 |
| *Cd1d1* | NM_007639 | +3.7 | -1.3 | +1.1 |
| *Cd36* | NM_007643 | +3.1 | +3.1 | +2.5 |
| *Cd40* | NM_011611 | +4.1 | -1.4 | -1.3 |
| *Cd169* | NM_011426 | +2.9 | +2.1 | +1.3 |
| *Cd300lf* | NM_145634 | +3.9 | +1.0 | +1.0 |
| *Cdc25c* | NM_004860 | +1.7 | -2.7 | -1.7 |
| *Cdc42* | NM_009861 | -4.6 | -1.3 | -1.2 |
| *Cdh1* | NM_009864 | -1.4 | -1.7 | -1.7 |
| *Cdk1* | NM-007659 | +1.0 | -1.5 | -1.6 |
| *Cdk1n1a* | NM_007669 | -1.6 | -1.0 | -1.5 |
| *Cdkn1b* | NM_009875 | -3.1 | +1.3 | -1.1 |
| *Chek2* | NM_016681 | +1.4 | -1.7 | +1.4 |
| *Clec14a* | NM_025809 | +2.3 | +1.3 | +1.2 |
| *Clip1* | NM_019765 | +2.1 | -1.3 | +1.4 |
| *Clip2* | NM_009990 | +1.0 | +1.3 | -1.0 |
| *Crp* | NM_007768 | +27.7 | +4.7 | +3.3 |
| *Ctnnb1* | NM_007614 | +1.4 | -1.3 | -1.2 |
| *Cxcl1* | NM_008176 | +6.4 | -1.6 | -1.3 |
| *Cxcl15* | NM_011339 | +2.7 | -1.6 | -1.8 |
| *Cxcr1* | NM_178241 | +2.8 | +1.0 | +1.2 |
| *Defb1* | NM_007843 | -2.2 | -3.5 | -2.6 |
| *Defb4* | NM_019728 | -1.6 | -2.3 | -2.3 |
| *Derl1* | NM_024207 | +1.1 | -1.1 | -1.8 |
| *Dnajc3* | NM_008929 | +1.7 | +1.2 | +1.0 |
| *Dram2* | NM_026013 | -2.2 | -1.6 | -1.3 |
| *Ecsit* | NM_012029 | +2.2 | +1.2 | +1.3 |
| *Edem1* | NM_138677 | +1.4 | +1.0 | -1.0 |
| *Egfr* | NM_007192 | -6.8 | -1.3 | -1.5 |
| *Egr1* | NM_007913 | +2.9 | -1.3 | -1.6 |
| *Elmo1* | NM_198093 | +1.8 | +1.4 | -1.1 |
| *Epcam* | NM_008532 | -1.3 | -1.4 | -2.1 |
| *Ern1* | NM_023913 | +2.0 | +1.1 | -1.3 |
| *Erp44* | NM_029572 | -2.1 | -1.4 | -1.2 |
| *Fas* | NM_007987 | +1.6 | +1.3 | -1.3 |
| *Fbxo6* | NM_015797 | +1.5 | +1.2 | -1.2 |
| *Fcgr4* | NM_144559 | +8.7 | +3.1 | +1.6 |
| *Fos* | NM_010234 | +2.3 | -1.1 | -1.1 |
| *Foxo3* | NM_019740 | -1.7 | -1.5 | -1.1 |
| *Gabarap* | NM_019749 | -2.7 | -1.1 | +1.0 |
| *Gabarapl2* | NM_026693 | -1.7 | -1.7 | +1.0 |
| *Gadd45a* | NM_007836 | +1.3 | -1.4 | +1.1 |
| *Ganab* | NM_008060 | +2.2 | -1.1 | -1.1 |
| *Ganc* | NM_172672 | +3.4 | +1.2 | -1.1 |
| *Grid2* | NM_008167 | +3.3 | -2.6 | -1.1 |
| *Gulp1* | NM_028450 | +2.8 | -1.1 | -1.1 |
| *Hdac6* | NM_0110413 | +2.8 | -1.1 | -1.1 |
| *Hif1a* | NM_010431 | -6.1 | -1.3 | -1.1 |
| *Htra2* | NM_019752 | +1.2 | -1.5 | -2.0 |
| *Il1a* | NM_010554 | -4.3 | -2.3 | -2.0 |
| *Il1b* | NM_008361 | -1.9 | -2.5 | -2.3 |
| *Il6* | NM_031168 | +1.0 | -6.8 | -2.3 |
| *Il23a* | NM_031252 | +3.0 | -2.2 | +1.3 |
| *Iqgap1* | NM_016721 | -1.3 | +1.0 | +1.0 |
| *Irf3* | NM_016849 | -2.8 | -1.1 | -1.1 |
| *Irf7* | NM_016850 | +3.1 | +2.2 | -1.0 |
| *Itgam* | NM_008401 | +1.1 | -1.2 | -1.3 |
| *Itgb2* | NM_008404 | +3.7 | +1.6 | +1.1 |
| *Jun* | NM_010591 | +1.3 | +1.2 | -1.4 |
| *Krt5* | NM_027011 | -1.5 | -1.8 | -1.1 |
| *Krt8* | NM_031170 | +6.2 | +7.6 | +1.1 |
| *Krt18* | NM_010664 | +2.3 | +3.0 | -1.1 |
| *Lgals3* | NM_010705 | -1.0 | -1.3 | -1.0 |
| *Lilra6* | NM_011090 | +1.4 | +1.5 | +1.5 |
| *Ly96* | NM_016923 | +1.5 | +1.6 | +1.0 |
| *Map2k1* | NM_008927 | +1.1 | +1.2 | -1.0 |
| *Marco* | NM_010766 | +2.8 | +1.8 | -1.1 |
| *Map3k5* | NM_008580 | -1.3 | +1.1 | -1.2 |
| *Mcl1* | NM_008562 | -1.1 | +1.1 | -1.1 |
| *Mcph1* | NM_173189 | -1.3 | -1.3 | -2.1 |
| *Mdm2* | NM_010786 | +1.1 | -1.2 | -1.1 |
| *Mmp10* | NM_019471 | +1.0 | +1.5 | -1.0 |
| *Naip 1* | NM_008670 | +2.6 | +3.1 | +1.2 |
| *Nanog* | NM_028016 | +2.1 | +2.5 | -1.4 |
| *Ncoa4* | NM_019744 | +1.2 | +1.6 | -1.1 |
| *Nlrp3* | NM_145827 | +1.0 | +1.6 | -1.2 |
| *Nlrp4e* | NM_001004194 | +1.8 | +2.0 | -1.6 |
| *Nlrp6* | NM_001081389 | +1.1 | +1.1 | -2.0 |
| *Nme1* | NM_008704 | +1.0 | -1.1 | -1.1 |
| *Nod1* | NM_172729 | +1.1 | +1.5 | +1.1 |
| *Nod2* | NM_145857 | -1.2 | +1.2 | -1.0 |
| *Nos2* | NM_010927 | -1.4 | +6.5 | +1.4 |
| *Notch1* | NM_008714 | -1.3 | -2.4 | -1.9 |
| *Nucb1* | NM_008749 | -1.3 | +1.5 | +1.0 |
| *Ogg1* | NM_010957 | +1.2 | +1.6 | -1.0 |
| *Parp2* | NM_009632 | -1.1 | +1.2 | -1.2 |
| *Pkca* | NM_011101 | +1.4 | +1.5 | -1.0 |
| *Pik3ca* | NM_008839 | +1.1 | +1.5 | -1.1 |
| *Plcb1* | NM_019677 | -1.3 | +1.1 | +1.0 |
| *Pld1* | NM_008875 | -1.0 | +1.2 | -1.0 |
| *Ppara* | NM_011144 | -1.2 | +1.3 | -1.5 |
| *Ppard* | NM_011145 | -1.2 | +1.1 | -1.4 |
| *Pparg* | NM_011146 | +2.2 | +1.9 | -1.0 |
| *Prtn3* | NM_011178 | +2.1 | +8.1 | +2.6 |
| *Ppargc1a* | NM_008904 | +1.4 | +1.4 | -1.3 |
| *Ptch1* | NM_008957 | +1.4 | -1.1 | -1.4 |
| *Ptk2* | NM_007982 | +1.7 | +1.7 | +1.1 |
| *Ptx3* | NM_008987 | +1.7 | +2.1 | +1.3 |
| *Rab5a* | NM_025887 | +1.0 | +1.2 | +1.1 |
| *Rab7* | NM_009005 | +1.2 | +1.4 | -1.0 |
| *Rab11a* | NM_017382 | -1.1 | -1.1 | -1.1 |
| *Rab24* | NM_009000 | -1.2 | -1.3 | -1.0 |
| *Rad17* | NM_011233 | -1.4 | +1.5 | -1.3 |
| *Rad52* | NM_011236 | +1.1 | +2.2 | +1.0 |
| *Raf1* | NM_029780 | +1.1 | +1.4 | -1.1 |
| *Relb* | NM_009046 | +1.8 | +2.3 | -1.3 |
| *Rhoa* | NM_016802 | -1.2 | -1.3 | -1.3 |
| *Ripk1* | NM_009068 | -1.2 | +1.0 | -1.0 |
| *Ripk2* | NM_138592 | -1.0 | -1.2 | -1.3 |
| *Rpn1* | NM_133933 | -1.1 | +1.3 | -1.2 |
| *Rock1* | NM_009071 | +1.4 | +1.7 | +1.0 |
| *Rps6ka5* | NM_153587 | +1.0 | +1.4 | -1.3 |
| *S100a7a* | NM_199422 | -2.0 | -1.5 | -2.0 |
| *S100a8* | NM_013650 | -4.6 | -1.9 | -2.0 |
| *S100a9* | NM_009114 | -3.8 | -1.4 | -1.6 |
| *S100b* | NM_009115 | +1.8 | +2.1 | -1.0 |
| *Sec22b* | NM_011342 | +1.2 | +1.7 | -1.0 |
| *Sec61a1* | NM_016906 | +1.2 | +1.8 | +1.1 |
| *Shc1* | NM_011368 | +1.3 | -1.0 | -1.1 |
| *Sod3* | NM_011435 | +1.4 | +1.3 | -1.2 |
| *Sos1* | NM_009231 | +1.1 | +1.2 | -1.2 |
| *Sra1* | NM_025291 | +1.0 | +1.1 | -1.1 |
| *Src* | NM_009271 | +1.5 | +1.8 | -1.2 |
| *Slpi* | NM_011414 | +1.9 | +1.1 | -1.4 |
| *Tab2* | NM_138667 | +1.1 | +1.2 | -1.1 |
| *Tap2* | NM_011530 | +1.0 | +1.7 | -1.4 |
| *Tapbp* | NM_009318 | +1.5 | +2.1 | -1.3 |
| *Tdgf1* | NM_011562 | +1.7 | +1.2 | -1.4 |
| *Terf2* | NM_009353 | +1.1 | +1.6 | -1.2 |
| *Tff3* | NM_011575 | +2.0 | +1.5 | +1.0 |
| *Tlr1* | NM_030682 | -1.0 | +1.8 | -1.6 |
| *Tlr2* | NM_011905 | -1.1 | -1.0 | -1.3 |
| *Tlr4* | NM_021297 | +1.0 | +1.1 | -1.3 |
| *Tmem57* | NM_025382 | -1.1 | +1.2 | -1.4 |
| *Tnf* | NM_013693 | -1.4 | -1.9 | -2.3 |
| *Trp53* | NM_011640 | -1.0 | +1.2 | -1.5 |
| *Tr53bp1* | NM_013735 | +1.0 | +1.3 | -1.1 |
| *Uggt1* | NM_198899 | +1.1 | +1.8 | +1.0 |
| *Xbp1* | NM_013842 | -1.2 | +1.7 | -1.0 |
| *Ywhab* | NM_018753 | +1.0 | +1.6 | -1.2 |
| *Zfp42* | NM_009556 | +1.5 | +1.6 | -1.1 |
