# Supplementary material for: Intravaginal poly-(D, L-lactic-co-glycolic acid)-(polyethylene glycol) drug-delivery nanoparticles induce pro-inflammatory responses with Candida albicans infection in a mouse model
Source: PLoS One. 2020 Oct 22;15(10):e0240789. doi: 10.1371/journal.pone.0240789 (PMC7580924; doi:10.1371/journal.pone.0240789)
Supplement: S1 File — (DOCX) [file pone.0240789.s002.docx]

Fig 1. Nanoparticle colocalizations

|  | PLGA**^+^** EPCAM**^+^** | | | | | |
| --- | --- | --- | --- | --- | --- | --- |
| PLGA | 7.28 | 9.50 | 7.39 | 7.33 | 7.65 | 7.31 |
| PLGA *+ Ca* | 12.71 | 10.32 | 10.34 | 15.71 | 19.54 | 8.46 |

|  | PLGA^+^F4/80^+^ | | | | | |
| --- | --- | --- | --- | --- | --- | --- |
| PLGA | 5.22 | 8.55 | 3.96 | 7.13 | 4.06 | 6.27 |
| PLGA + *Ca* | 10.01 | 12.92 | 10.79 | 15.75 | 15.30 | 9.05 |

|  | PLGA^+^CD11c^+^ | | | | | |
| --- | --- | --- | --- | --- | --- | --- |
| PLGA | 4.18 | 8.12 | 9.30 | 8.80 | 3.38 | 5.69 |
| PLGA + *Ca* | 8.48 | 2.15 | 1.82 | 13.02 | 8.99 | 8.86 |
|  |  |  |  |  |  |  |

Fig 2A. Persistence of nanoparticle fluorescence (RFU x 10^4^) in vaginal tracts of mice after challenge.

| 2 Hour | | | | | | |
| --- | --- | --- | --- | --- | --- | --- |
| Vag wash | 6.127665 | 5.043408 | 13.75953 | 8.331820 | 1.729311 | 3.179724 |
| Cells | 2.540655 | 2.121206 | 2.127607 | 2.151746 | 2.244299 | 3.128668 |
| 6 Hour | | | | | | |
| Vag wash | 2.114232 | 2.243670 | 2.811974 | 1.891852 | 1.703095 | 4.427731 |
| Cells | 2.006270 | 2.232535 | 2.271194 | 1.994915 | 2.035297 | 8.390997 |
| 18 Hour | | | | | | |
| Vag wash | 3.990991 | 9.376007 | 17.944910 | 19.910040 | 5.7705160 |  |
| Cells | 0.855785 | 0.849393 | 0.857165 | 0.8550662 | 0.8636169 | 0.832715 |

Fig 2B. Persistence of *C. albicans* CFU counts (CFU/mL X 10^3^) in vaginal tracts of mice after challenge.

| 2 Hour | | | | | | |
| --- | --- | --- | --- | --- | --- | --- |
| Vag wash |  |  | 2 | 2 | 4 | 4 |
| Cells | 1.5 | 1.5 | 1.5 | 1.5 | 1.5 | 1.5 |
| 6 Hour | | | | | | |
| Vag wash | 1 | 1 | 2 |  | 1 | 1 |
| Cells |  | 0.4 | 0.34 | 0.23 | 0.014 | 0.003 |
| 18 Hour | | | | | | |
| Vag wash | 0.52 |  | 0.17 | 0.86 | 0.89 |  |
| Cells | 0.038 | 0.002 | 0.01 | 0.034 | 0.071 | 0.086 |

Fig 3A. Detection of nanoparticles fluorescence (RFU x 10^3^) in organs.

| Liver | | | | | | |
| --- | --- | --- | --- | --- | --- | --- |
| Vag wash | 17.388 | 0.633 | 14.146 | 14.085 |  | 18.05 |
| Cells | 11.313 | 15.531 | 16.612 | 17.066 | 12.855 | 14.815 |
| Spleen | | | | | | |
| Vag wash | 0.062 | 2.465 | 0.025 | 0.038 | 2.433 | 0.484 |
| Cells | 0.085 | 0.312 | 0.799 | 0.874 | 0.698 | 1.089 |
| Kidney | | | | | | |
| Vag wash | 0.841 | 0.62 | 2.52 | 2.541 | 2.474 | 2.421 |
| Cells | 2.713 | 0.158 | 4.164 | 6.733 | 2.224 | 4.519 |
| Heart | | | | | | |
| Vag wash | 0 | 0 | 0 | 0 | 0 | 0 |
| Cells |  | 7.189 | 9.7e-002 | 6.695 | 6.28 |  |
| Lung | | | | | | |
| Vag wash | 15.2 | 5.681 | 4.026 | 12.254 | 6.39 | 8.048 |
| Cells | 7.718 |  | 6.624 | 6.695 | 6.28 | 7.054 |

|  |  |
| --- | --- |

Fig 3B. Detection of *C. albicans* in organs (CFU/mL x10^3^).

| Liver | | | | | | |
| --- | --- | --- | --- | --- | --- | --- |
| Vag wash | 1.2 | 0.12 | 0.06 | 1.2 | 0.48 | 0.66 |
| Cells | 0.6 | 4.26 | 2.28 | 3.3 | 0.24 | 1.44 |
| Spleen | | | | | | |
| Vag wash | 0.12 |  |  | 0.54 | 1.08 |  |
| Cells | 0.18 | 0.24 |  | 0.66 | 0.56 |  |
| Kidney | | | | | | |
| Vag wash | 0.12 | 0.6 |  |  | 0.12 | 0.12 |
| Cells |  | 0.18 | 0.3 | 0.3 | 0.18 |  |
| Heart | | | | | | |
| Vag wash | 0.72 | 0.12 | 0.84 |  | 1.8 | 0.18 |
| Cells |  | 1.32 | 2.28 | 3.36 | 3.24 |  |
| Lung | | | | | | |
| Vag wash |  |  | 5.34 | 3 | 0.42 | 0.36 |
| Cells | 2.16 | 1.62 | 1.98 | 2.76 | 2.34 |  |

Fig 4A. Serum LDH (mU/mL X 10^2^)

| Control | *Ca* | PLGA | PLGA + *Ca* |
| --- | --- | --- | --- |
| 25.1 | 58.0 | 30.67 | 123.3 |
| 22.0 | 56.5 | 31.2 | 122.5 |
| 23.4 | 57.6 | 33.5 | 124.0 |
| 22.6 | 54.9 | 32.1 | 121.0 |
| 21.7 | 58.3 | 32.5 | 125.0 |
| 23.5 | 54.9 | 31.1 | 123.0 |

Fig 4B. Apoptosis as cleaved PARP, % Positive cells.

| Ctrl | *Ca* | PLGA | PLGA + *Ca* |
| --- | --- | --- | --- |
| 11.63 | 7.64 | 58.77 | 76.52 |
| 8.13 | 29.45 | 52.86 | 55.92 |
| 36.46 | 21.69 | 66.09 | 72.34 |
| 30.68 |  | 51.01 | 72.31 |
| 21.75 |  | 37.17 | 72.44 |
| 18.61 |  |  |  |

Fig 4C. DNA damage as γh2AX % positive cells.

| Ctrl | *Ca* | PLGA | PLGA + *Ca* |
| --- | --- | --- | --- |
| 8.74 | 31.81 | 39.14 | 38.07 |
| 13.31 | 36.25 | 53.39 | 26.90 |
| 11.33 | 27.27 | 63.48 | 34.98 |
| 13.86 |  | 72.56 | 32.38 |
| 10.27 |  | 55.75 | 28.21 |
| 12.43 |  |  |  |

Fig 4D. Mitochondrial depolarization (% positive cells).

| Ctrl | *Ca* | PLGA | *Ca* + PLGA |
| --- | --- | --- | --- |
| 5.65 | 3.31 | 81.24 | 13.48 |
| 5.16 | 6.42 | 45.78 | 96.40 |
| 7.96 | 6.20 | 38.56 | 15.40 |
| 3.87 | 6.95 | 58.20 | 9.35 |
| 4.78 | 26.2 | 44.30 | 56.51 |
| 4.76 | 41.2 | 52.38 | 7.64 |

Fig 4E. Oxidative stress as total ROS (RFU X 10^4^).

| Ctrl | *Ca* | PLGA | PLGA + *Ca* |
| --- | --- | --- | --- |
| 0.060824 | 3.901652 | 0.280106 | 0.1929175 |
| 0.080235 | 4.299073 | 0.062736 | 2.2270220 |
| 0.099342 | 1.232665 | 0.209013 | 2.8481960 |
| 1.814640 | 2.197682 | 0.314035 | 1.1766750 |
| 1.332170 |  | 0.291445 | 2.6910460 |
| 1.121040 |  | 0.163022 | 2.0202940 |

Fig 4F. Autophagy data (% Positive cells).

| Ctrl | *Ca* | PLGA | *Ca* + PLGA |
| --- | --- | --- | --- |
| 5.89 | 6.28 | 25.62 | 37.17 |
| 8.74 | 12.01 | 6.51 | 31.68 |
| 8.00 | 17.77 | 5.67 | 23.04 |
| 9.70 | 1.60 | 10.00 | 14.00 |
| 10.42 | 10.47 | 9.12 | 20.64 |
| 4.51 | 4.69 | 11.21 | 17.14 |

Fig 4G. ER stress as protein aggresomes, (RFU X 10^5^)

| Ctrl | *Ca* | PLGA | PLGA + *Ca* |
| --- | --- | --- | --- |
| 0.19850 | 1.19781 | 1.05567 | 0.97301 |
| 0.49773 | 0.96161 | 0.60724 | 0.75732 |
| 0.79451 | 1.00678 | 0.4634 | 0.94134 |
| 0.57296 | 1.10216 | 0.51012 | 0.74225 |
|  |  | 0.77177 | 0.86857 |
|  |  | 0.83108 | 0.75895 |

Fig 5A. Serum IL-1β (pg/mL)

| Ctrl | *Ca* | PLGA | *Ca*+ PLGA |
| --- | --- | --- | --- |
| 31.5 | 80.0 | 222.5 | 176.0 |
| 30.0 | 99.0 | 256.5 | 220.0 |
| 13.5 | 23.0 | 182.0 | 284.5 |
| 32.0 | 4.02 | 149.0 | 262.0 |
| 31.0 | 77.0 | 222.5 | 176.0 |
|  |  |  |  |

Fig 5B. Vaginal wash IL-1β (pg/mL)

| Ctrl | *Ca* | PLGA | *Ca* + PLGA |
| --- | --- | --- | --- |
| 37.66 | 313.99 | 425.47 | 74.27 |
| 180.62 | 530.68 | 1065.53 | 74.27 |
| 13.68 | 341.27 | 229.10 | 223.86 |
|  | 61.52 | 1360.03 | 471.31 |
|  | 478.84 | 123.63 |  |
|  |  |  |  |

Fig 5C. Serum IL-6 (pg/mL)

| Ctrl | *Ca* | PLGA | *Ca* + PLGA |
| --- | --- | --- | --- |
|  |  |  |  |
| 76.5 | 368.0 | 633.0 | 622.0 |
| 133.0 | 200.0 | 355.0 | 372.0 |
| 179.5 | 248.0 | 398.0 | 533.0 |
| 108.0 | 340.0 | 303.0 | 392.5 |
|  |  |  |  |

Fig 5D. Serum TNFα (pg/mL)

| Ctrl | *Ca* | PLGA | *Ca* + PLGA |
| --- | --- | --- | --- |
| 26.0 | 120.0 | 135.0 | 204.5 |
| 64.5 | 97.0 | 71.5 | 149.0 |
| 20.0 | 35.0 | 155.0 | 133.0 |
| 54.0 | 114.0 | 145.0 | 155.0 |
| 52.5 | 71.0 |  |  |
|  |  |  |  |

Fig 6A. PMN chemotaxis, Cells X 10^5^; CCL5 negative control, CXCL2 positive control

| CCL5 | CXCl2 | *Ca* | PLGA | PLGA + *Ca* |
| --- | --- | --- | --- | --- |
| 0 | 1.30 | 1.18 | 3.88 | 1.86 |
| 0 | 1.25 | 1.34 | 5.40 | 2.09 |
| 0 | 1.12 | 0.83 | 4.26 | 1.50 |
| 0 | 1.33 | 1.38 | 2.90 | 1.48 |
| 0 | 1.41 | 1.51 | 4.05 | 1.51 |
| 0 | 1.50 |  | 3.11 |  |

Fig 6B. BMDC chemotaxis, Cells X 10^5^; CCL5 neg ctrl, CCL21 positive control

| Ctrl | CCL5 | CCL21 | *Ca* | PLGA | PLGA + *Ca* |
| --- | --- | --- | --- | --- | --- |
| 0 | 0 | 1.60 | 3.41 | 2.70 | 2.92 |
| 0 | 0 | 2.13 | 4.20 | 3.32 | 3.21 |
| 0 | 0 | 1.35 | 4.10 | 1.62 | 5.04 |
| 0 | 0 | 1.20 | 4.25 | 2.10 | 3.11 |
| 0 | 0 | 1.34 | 3.46 | 2.70 | 3.62 |
| 0 | 0 | 1.52 | 4.70 |  |  |
