# Supplementary material for: Intravaginal poly-(D, L-lactic-co-glycolic acid)-(polyethylene glycol) drug-delivery nanoparticles induce pro-inflammatory responses with Candida albicans infection in a mouse model
Source: PLoS One. 2020 Oct 22;15(10):e0240789. doi: 10.1371/journal.pone.0240789 (PMC7580924; doi:10.1371/journal.pone.0240789)

Fig 1 Plots showing gates used to determine %POS cells values based on the positive control.

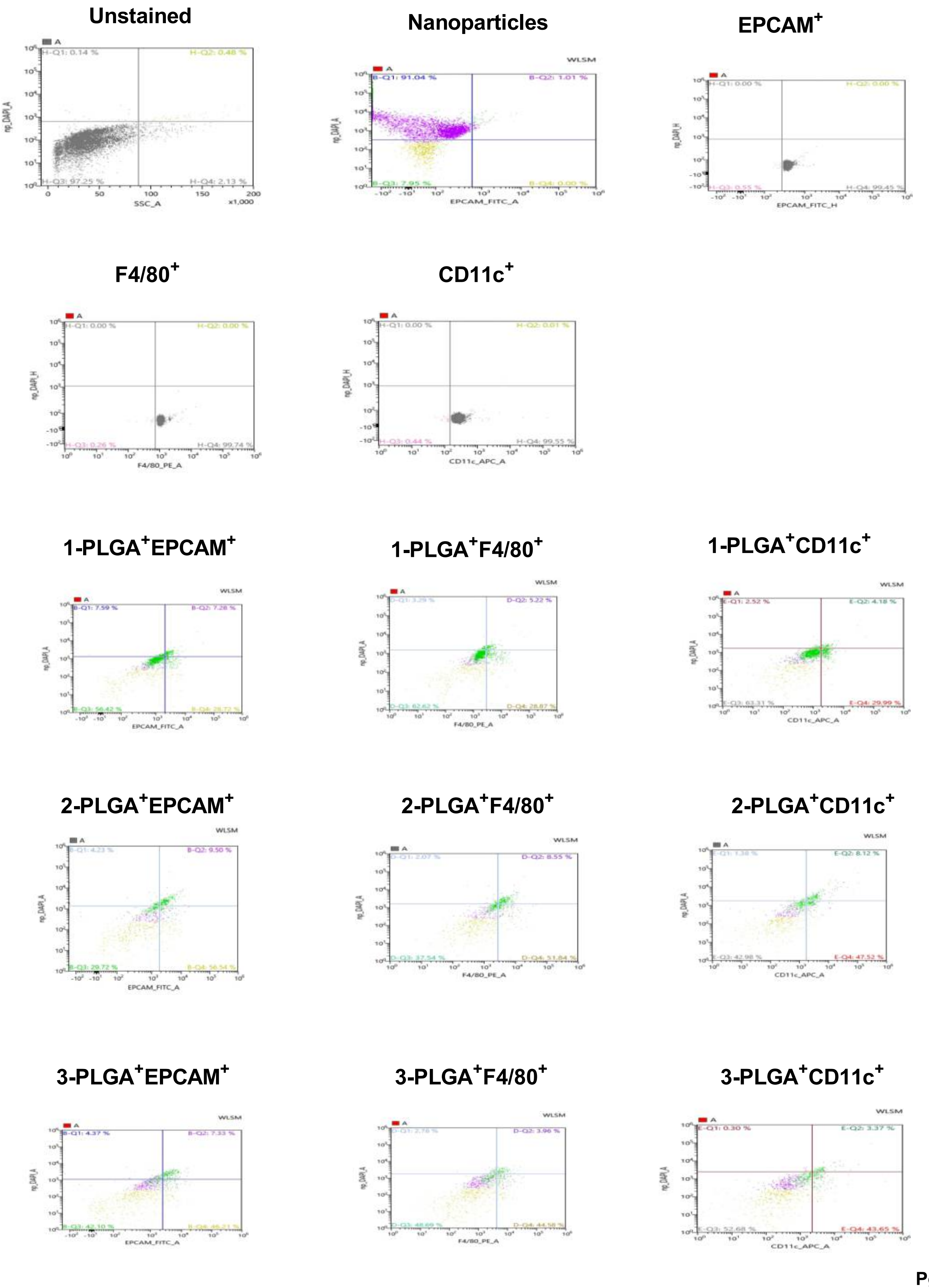

Fig 1 Plots showing gates used to determine %POS cells values based on the positive control.

4-PLGA⁺EPCAM⁺

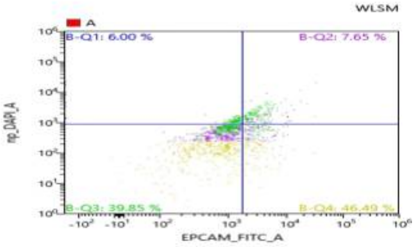

4-PLGA⁺F4/80⁺

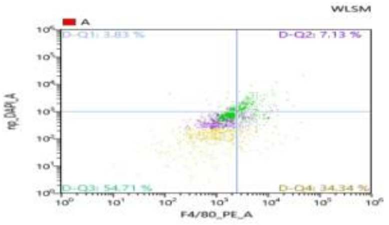

4-PLGA⁺CD11c⁺

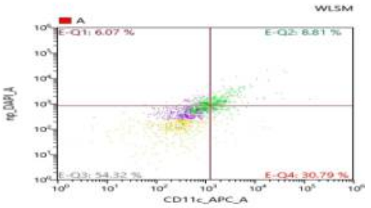

5-PLGA⁺EPCAM⁺

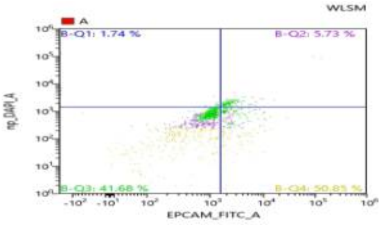

5-PLGA⁺F4/80⁺

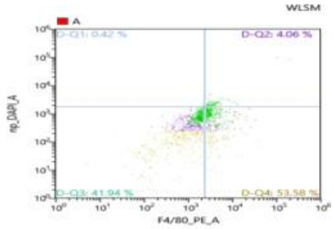

5-PLGA⁺CD11c⁺

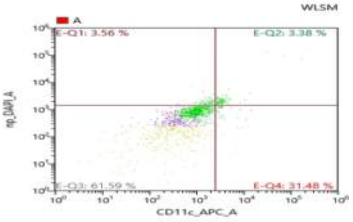

6-PLGA⁺EPCAM⁺

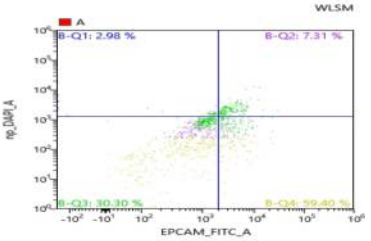

6-PLGA⁺F4/80⁺

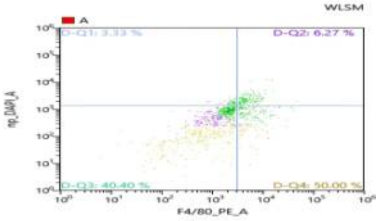

6-PLGA⁺CD11c⁺

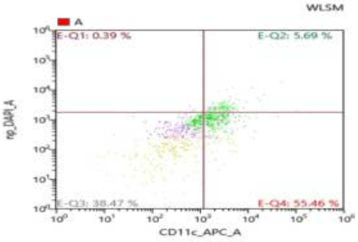

1-PLGA⁺*Ca*⁺EPCAM⁺

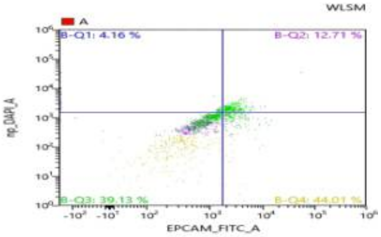

1-PLGA⁺*Ca*⁺F4/80⁺

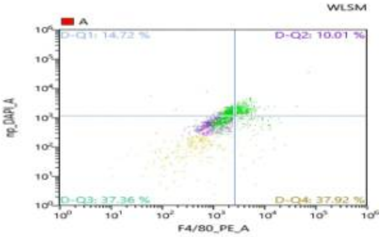

1-PLGA⁺*Ca*⁺CD11c⁺

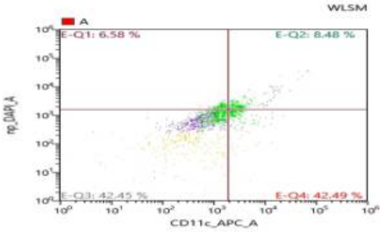

2-PLGA⁺*Ca*⁺EPCAM⁺

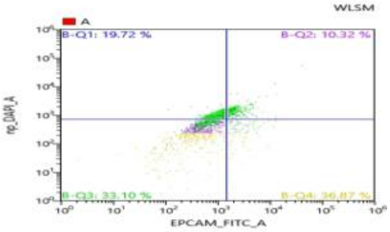

2-PLGA⁺*Ca*⁺F4/80⁺

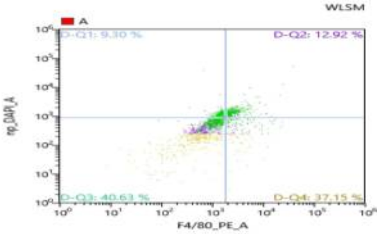

2-PLGA⁺*Ca*⁺CD11c⁺

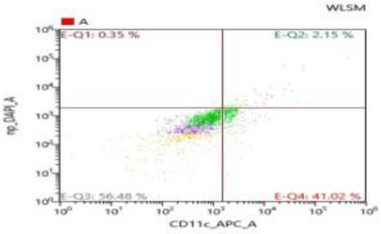

Fig 1 Plots showing gates used to determine %POS cells values based on the positive control.

3-PLGA⁺*Ca*⁺EPCAM⁺

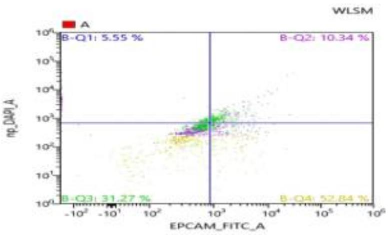

3-PLGA⁺*Ca*⁺F4/80⁺

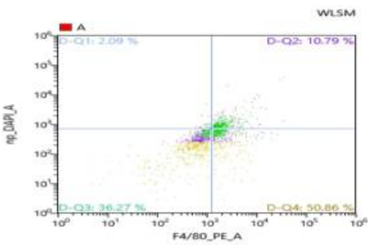

3-PLGA⁺*Ca*⁺CD11c⁺

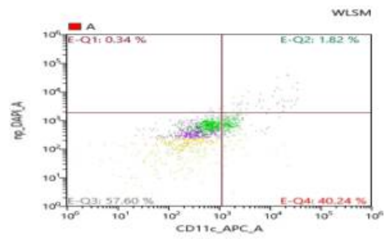

4-PLGA⁺*Ca*⁺EPCAM⁺

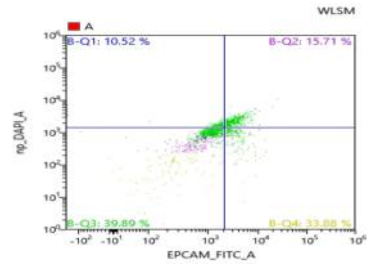

4-PLGA⁺*Ca*⁺F4/80⁺

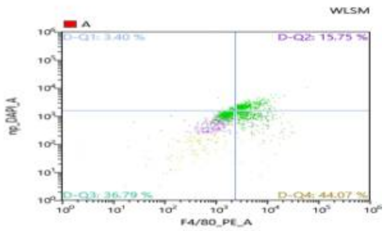

4-PLGA⁺*Ca*⁺CD11c⁺

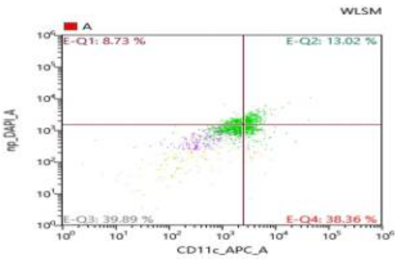

5-PLGA⁺*Ca*⁺EPCAM⁺

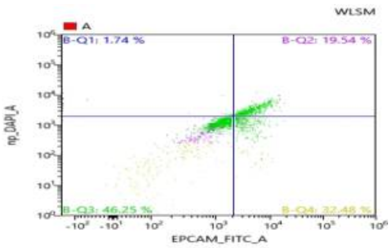

5-PLGA⁺*Ca*⁺F4/80⁺

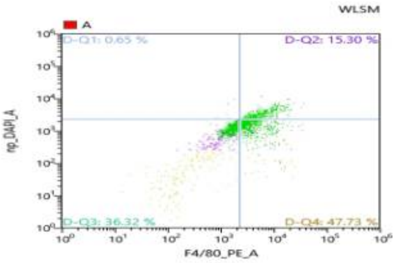

5-PLGA⁺*Ca*⁺CD11c⁺

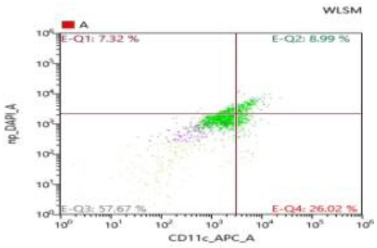

6-PLGA⁺*Ca*⁺EPCAM⁺

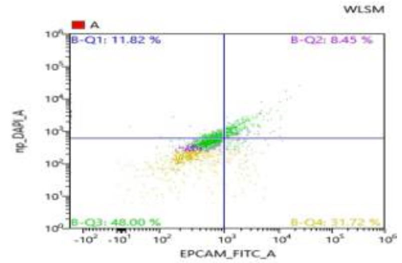

6-PLGA⁺*Ca*⁺F4/80⁺

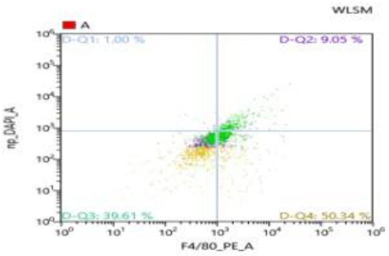

6-PLGA⁺*Ca*⁺CD11c⁺

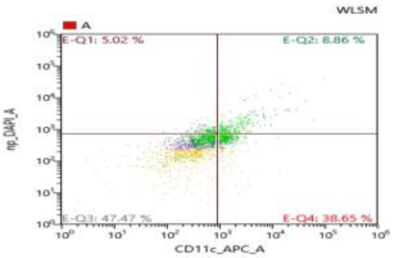

Fig 4B Histograms showing gates from the positive control to determine %cPARP results.

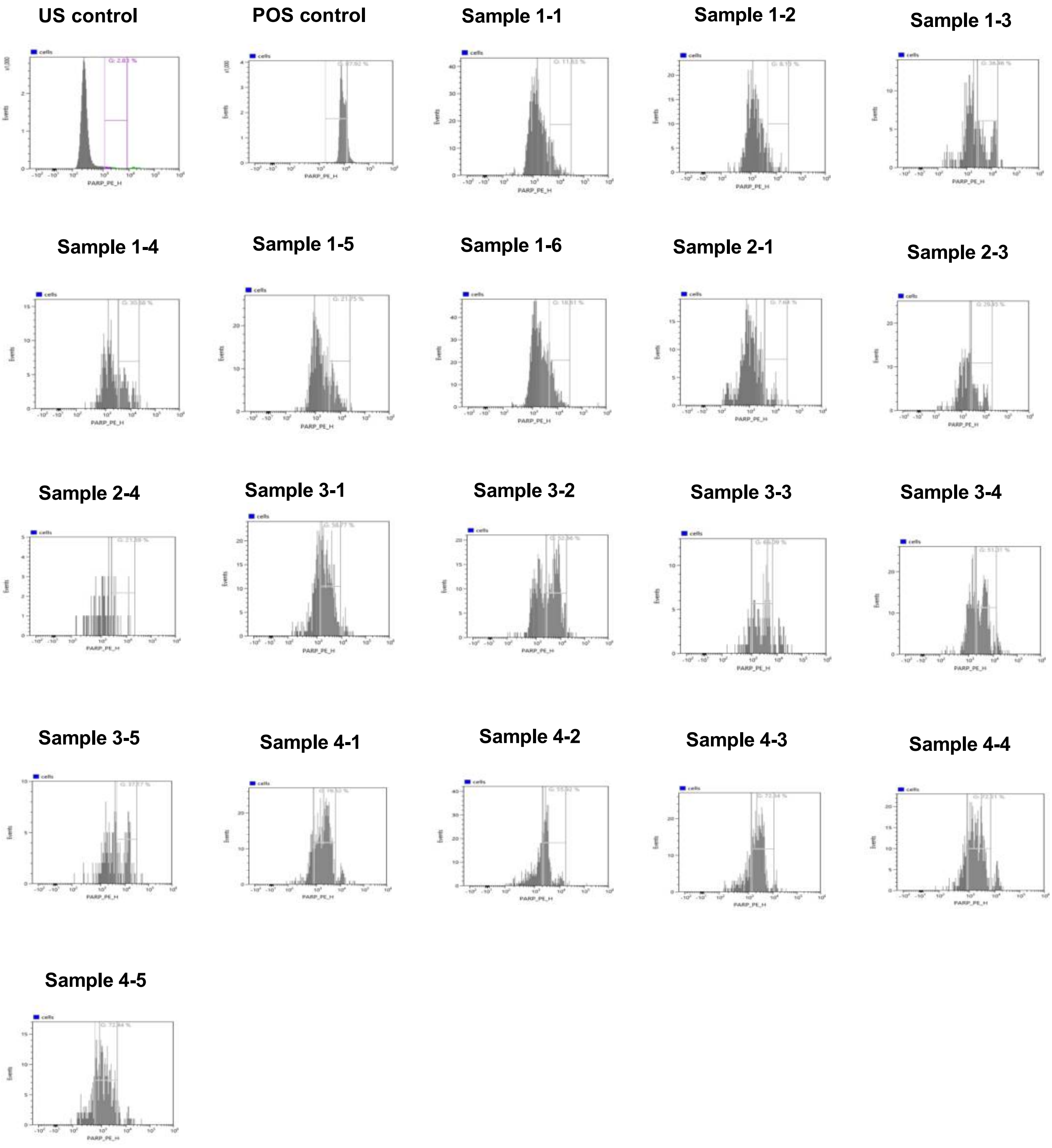

Fig 4C Histograms showing gates used to determine % Positive Cells values based on the positive control.

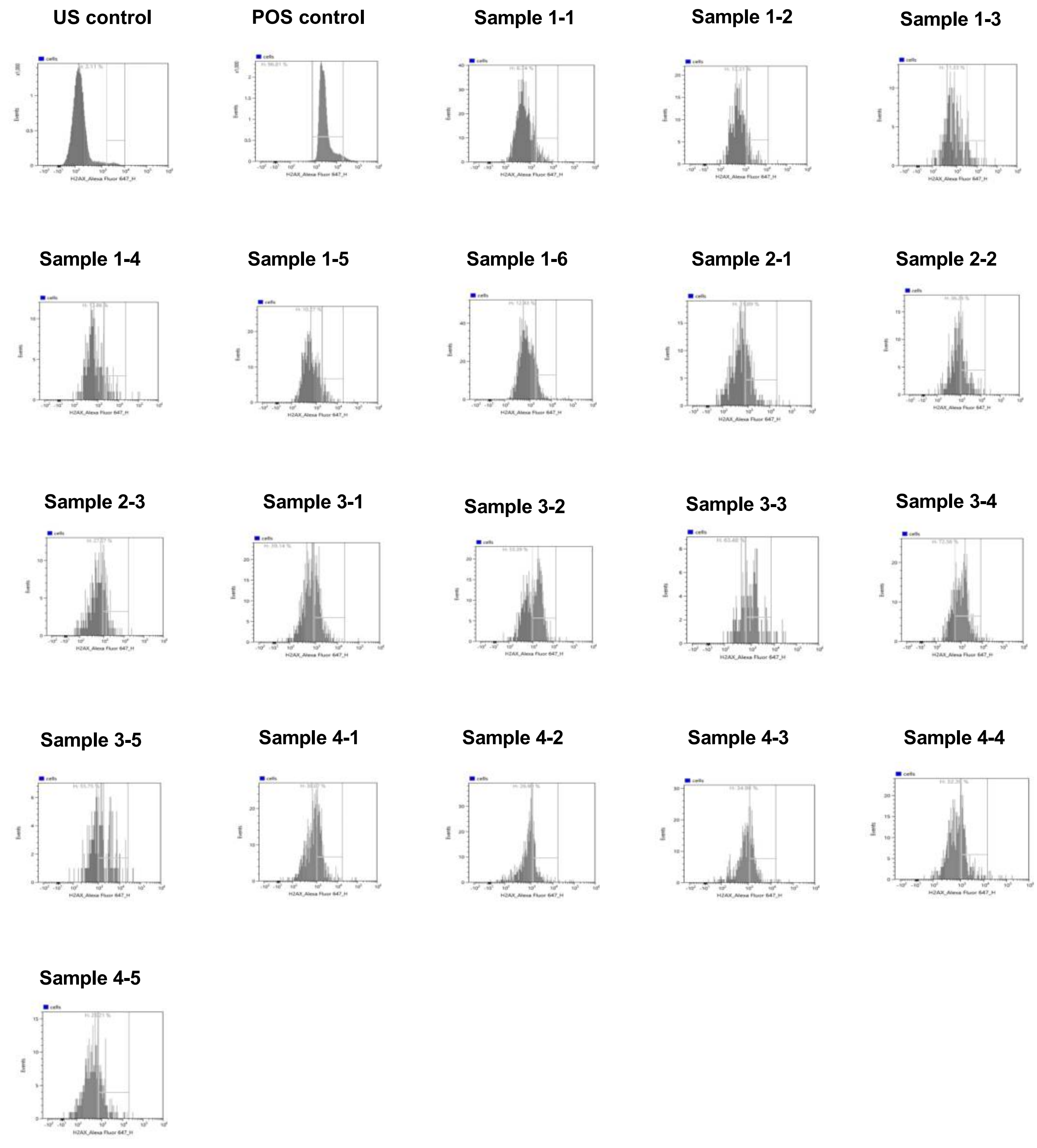

Fig 4D Histograms showing gates used to determine % DiIC$_1$(5)^{Low} Cells values based on the positive control.

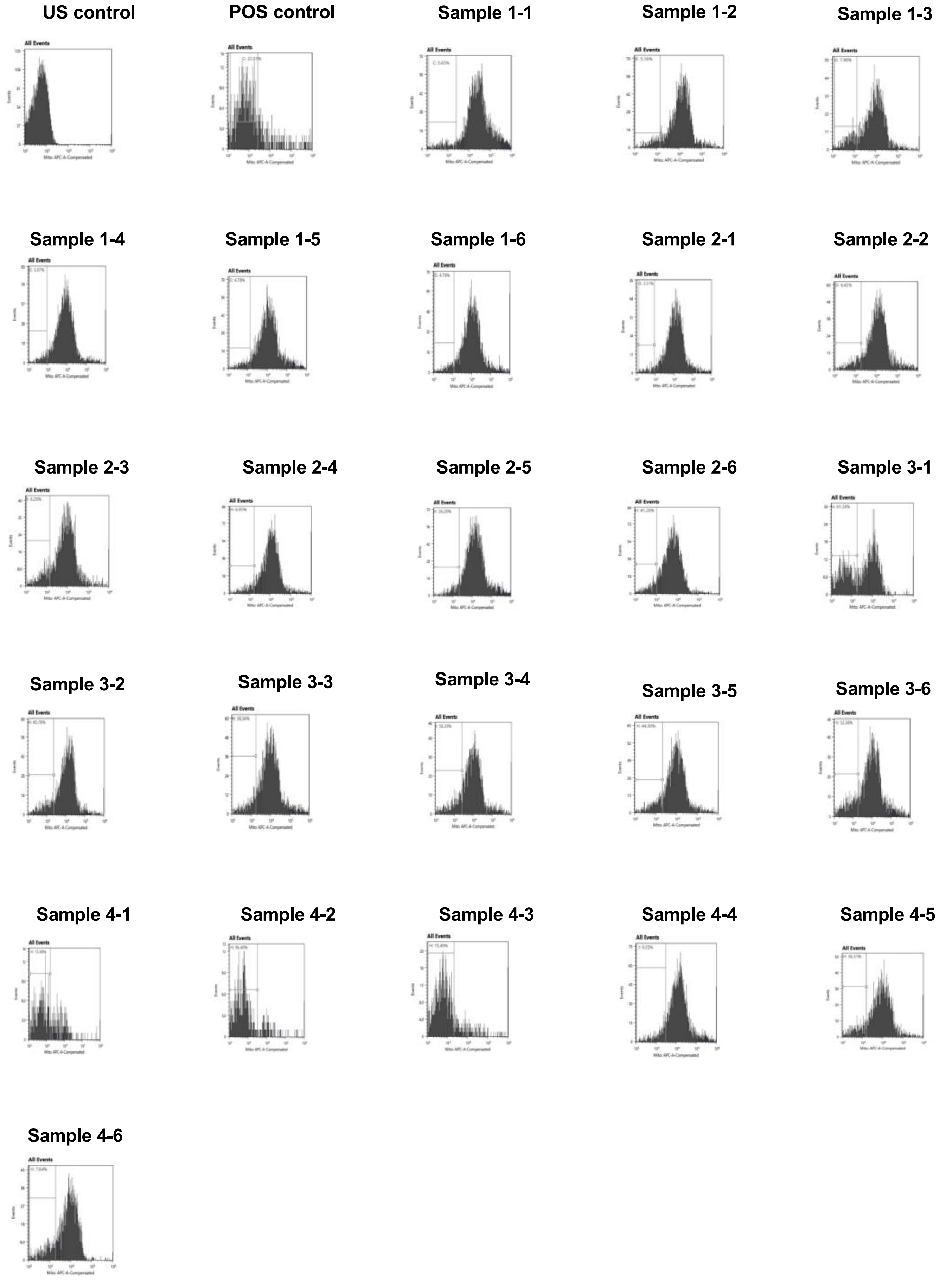

Fig 4E Histograms showing gates used to determine RFI values based on the positive control.

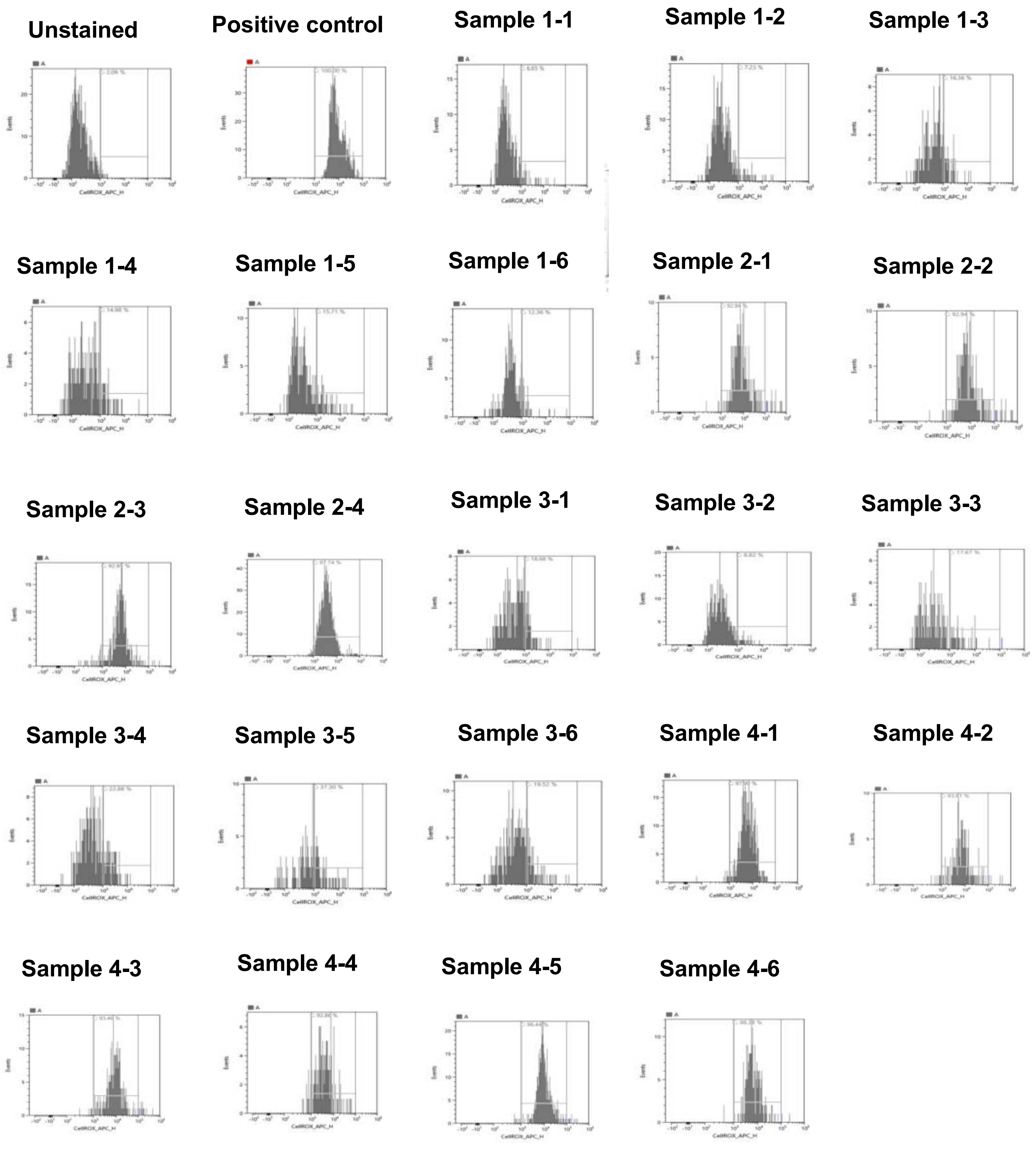

Fig 4F Histograms showing gates used to determine % Positive Cells values based on the positive control.

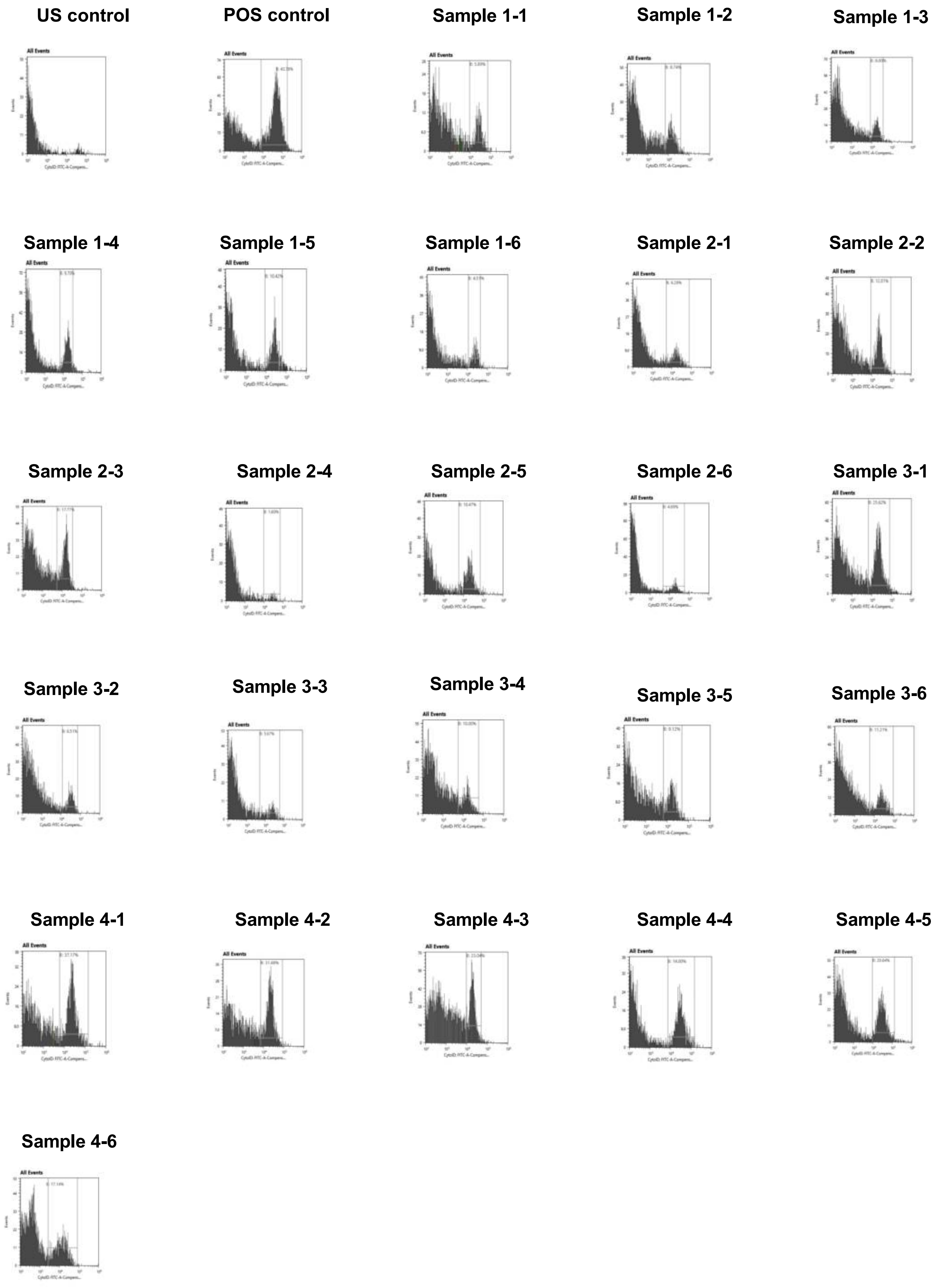

Fig 4G Histograms showing gates used to determine RFI values based on the positive control.

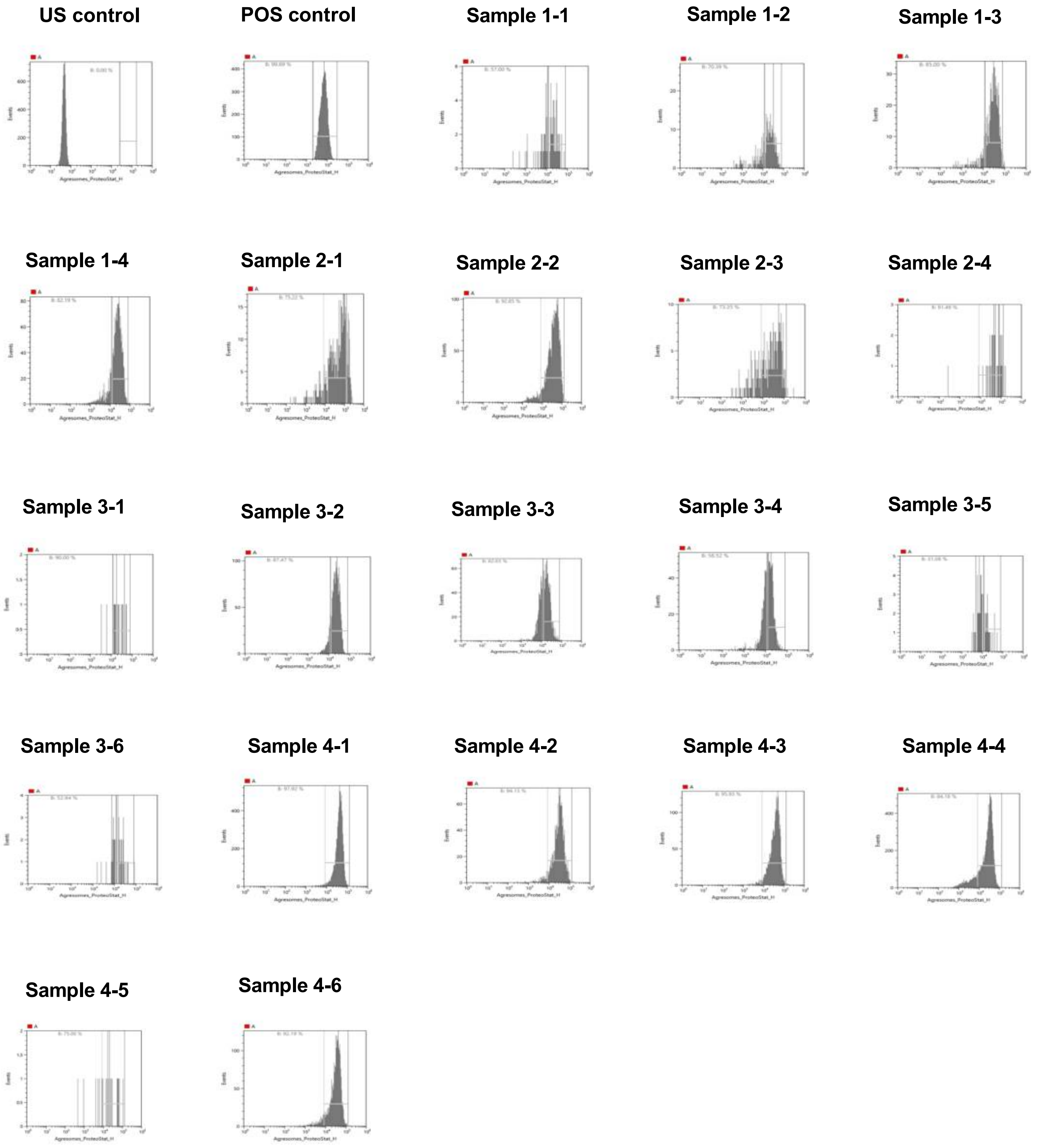

Supplement: S2 File — (PDF) [file pone.0240789.s003.pdf]
